# Using Machine Learning and Smartphone and Smartwatch Data to Detect Emotional States and Transitions: Exploratory Study

**DOI:** 10.2196/17818

**Published:** 2020-09-29

**Authors:** Madeena Sultana, Majed Al-Jefri, Joon Lee

**Affiliations:** 1 Data Intelligence for Health Lab Cumming School of Medicine University of Calgary Calgary, AB Canada; 2 Department of Community Health Sciences Cumming School of Medicine University of Calgary Calgary, AB Canada; 3 Department of Medicine Cumming School of Medicine University of Calgary Calgary, AB Canada; 4 Department of Cardiac Sciences Cumming School of Medicine University of Calgary Calgary, AB Canada

**Keywords:** mHealth, mental health, emotion detection, emotional transition detection, spatiotemporal context, supervised machine learning, artificial intelligence, mobile phone, digital biomarkers, digital phenotyping

## Abstract

**Background:**

Emotional state in everyday life is an essential indicator of health and well-being. However, daily assessment of emotional states largely depends on active self-reports, which are often inconvenient and prone to incomplete information. Automated detection of emotional states and transitions on a daily basis could be an effective solution to this problem. However, the relationship between emotional transitions and everyday context remains to be unexplored.

**Objective:**

This study aims to explore the relationship between contextual information and emotional transitions and states to evaluate the feasibility of detecting emotional transitions and states from daily contextual information using machine learning (ML) techniques.

**Methods:**

This study was conducted on the data of 18 individuals from a publicly available data set called ExtraSensory. Contextual and sensor data were collected using smartphone and smartwatch sensors in a free-living condition, where the number of days for each person varied from 3 to 9. Sensors included an accelerometer, a gyroscope, a compass, location services, a microphone, a phone state indicator, light, temperature, and a barometer. The users self-reported approximately 49 discrete emotions at different intervals via a smartphone app throughout the data collection period. We mapped the 49 reported discrete emotions to the 3 dimensions of the pleasure, arousal, and dominance model and considered 6 emotional states: discordant, pleased, dissuaded, aroused, submissive, and dominant. We built general and personalized models for detecting emotional transitions and states every 5 min. The transition detection problem is a binary classification problem that detects whether a person’s emotional state has changed over time, whereas state detection is a multiclass classification problem. In both cases, a wide range of supervised ML algorithms were leveraged, in addition to data preprocessing, feature selection, and data imbalance handling techniques. Finally, an assessment was conducted to shed light on the association between everyday context and emotional states.

**Results:**

This study obtained promising results for emotional state and transition detection. The best area under the receiver operating characteristic (AUROC) curve for emotional state detection reached 60.55% in the general models and an average of 96.33% across personalized models. Despite the highly imbalanced data, the best AUROC curve for emotional transition detection reached 90.5% in the general models and an average of 88.73% across personalized models. In general, feature analyses show that spatiotemporal context, phone state, and motion-related information are the most informative factors for emotional state and transition detection. Our assessment showed that lifestyle has an impact on the predictability of emotion.

**Conclusions:**

Our results demonstrate a strong association of daily context with emotional states and transitions as well as the feasibility of detecting emotional states and transitions using data from smartphone and smartwatch sensors.

## Introduction

The emotional states of individuals may change frequently over time. Research has demonstrated the potential of recording daily emotional states and moods in health and well-being, including the early diagnosis of mental illness and disorders [1-3]. However, the process of recording emotional states and moods largely depends on active self-reports less frequently than daily. However, with the unprecedented rise of smartphones and wearable devices as well as the advancement in built-in sensors within these devices, it is possible to passively collect multimodal data from people’s everyday lives at a much higher frequency. The self-reporting problem of personal health tracking can therefore be solved to a great extent by leveraging machine learning (ML) algorithms on the myriad of data collected by smartphones and wearables.

Predicting and monitoring mental health illnesses and diseases such as depression, bipolar disorder, Alzheimer disease, and schizophrenia via smartphones and wearable sensors have been an active area of research over the last few years. Research has been conducted in the quest for gold standard digital biomarkers that can be collected through consumer-grade smartphones and wearable sensors (eg, accelerometer, audio, location, phone log, sound features, etc) to detect mental health disorders in the early stages [4,5]. It is evident that mobility patterns, location variations, and phone usage patterns captured by smartphones can aid in identifying patients with mental health illnesses and disorders [1,2,6-9]. Early detection of depressive symptoms by applying deep neural networks and ML techniques to self-reported contextual data through smartphones obtained promising results [8]. However, the aforementioned apps were designed from disease and illness perspectives and did not consider the automated detection of regular emotional states and transitions in everyday life.

The association between everyday mood, emotion, and well-being and sensed data via smartphones and wearables has been studied recently. For example, Helbich [10] found an association of people’s mental well-being with the neighborhood they live in, the places they visit, and the environmental exposure they experience. In a similar study by Sandstrom et al [11], subjects reported emotional pleasantness in a societal environment, whereas positive and negative arousal at work. A daily mood assessment tool was proposed by Ma et al [12] that utilized mobile phone sensor data such as location, audio, text messages, accelerometer, and light to classify mood. However, this study considered limited contextual parameters, and the subjective variability as well as transitions of emotional states remained uninvestigated. The prediction of the Ecological Momentary Assessment scores from smartphone data such as text messages, screen time, app usage, accelerometer, and phone camera have been studied by Asselbergs et al [13], who reported a promising but lower prediction accuracy than naive benchmark approaches. Studies have also shown an association between mobile sensor data (eg, phone usage, motion, conversation, mobility, screen time, and skin conductance) and the academic performance and mental health conditions of college students [14,15]. Budner et al [16] classified 9 emotional states in 2 dimensions (pleasure and activation) of the circumplex model by applying a random forest on the smartwatch sensor data such as motion, heart rate, light level, GPS coordinates, day of the week, humidity, air pressure, cloudiness, and windiness. In a similar study, an ML-based model was proposed by Zhang et al [17] to recognize compound emotional states in pleasure and arousal dimensions from smartphone data (eg, microphone, accelerometer, GPS, text message, phone call, app usage). Promising results have been obtained in some recent works for daily mood and stress forecasting [3,18,19], where deep neural networks were applied to physiological, personality traits, and sensed data acquired from a large-scale global population using smartphones and wearables.

Despite encouraging results and progress, gaps in the literature include the lack of research on the association between emotional transition, sensed data, and contextual information; subjective variability in classification performance; and feasibility of frequent emotional state and transition detection. In addition, the majority of the previous studies are based on the circumplex model of affect [20], which considers only the pleasure and arousal dimensions (also known as *core affect*). However, research has demonstrated that considering all the 3 dimensions can facilitate a better understanding and interpretation of persons’ emotional states [21]. Therefore, the third dimension of emotional states—dominance—needs to be included in emotion recognition research.

In this study, we aim to fill the aforementioned gaps. Our main objective is to study the feasibility of detecting emotional states and transitions every 5 min by applying ML to the data acquired from smartphone and smartwatch sensors. Our study includes all the 3 dimensions of emotional states (pleasure, arousal, and dominance [PAD]) as well as the variability of interpersonal data. The remainder of this paper is organized as follows. The Methods section describes the methodology followed in our study along with an overview of the data set description and preparation. The Results section shows the results obtained for emotion transition and detection tasks for both general and personalized models. The Discussion section presents the results with a deeper analysis of the features.

## Methods

### The Data Set

We obtained data from a publicly available data set called ExtraSensory [22]. This data set was collected by the researchers of the University of California, San Diego (UCSD), in 2015 to 2016 for automated context labeling from signals captured via a wide range of smartphone and smartwatch sensors such as an accelerometer, a gyroscope, a magnetometer, a compass, location services, audio, phone state, light, air pressure, humidity, and temperature [23]. It contains data from 60 subjects in free-living conditions, who were mainly students (both undergraduate and graduate) and research assistants at UCSD. The sensor data were collected every minute, and the contextual data were self-reported at different intervals by the users. This data set also contains optionally self-reported discrete emotions at different time intervals. There were a total of 49 different discrete emotions (eg, active, calm, happy, sleepy, etc) that were reported by the subjects and the interval varied from 1 min to several days. Researchers processed and cleaned the self-reported data by combining various sources of information such as location and other labels [23] to make them reliable. Both the raw and cleaned versions of self-reported data are available. We used the cleaned version in this study.

### The Pleasure, Arousal, and Dominance Model

The PAD model was developed by Mehrabian and Russell [24] in 1974 to assess individuals’ psychological responses to environmental perception and experience. Persons’ emotional states can be perceived in 3 basic dimensions: pleasure, arousal, and dominance. Pleasure is the dimension of positive or negative feelings [24]. Arousal represents states of mental responsiveness [25]. Dominance is the perceptual cognitive dimension of the feeling influenced or controlled [25]. Our study includes all the 3 dimensions of emotional states.

### Data Preparation

#### Inclusion and Exclusion

In this exploratory study, we aimed to apply ML in the 2 setups. First, we built personalized models using each person’s data to analyze the impact of variability across individuals. Second, we built generalized models using data from multiple individuals and validated them using data from other individuals who were left out during training. Although 37 subjects in the ExtraSensory [22] data set reported their emotions at some points during data collection, only 18 of them had more than 1000 samples and less than 90% missing data. Therefore, we considered these 18 subjects in our study. We considered all signals collected from the smartphone and smartwatch sensors, timestamps, latitudes, and longitudes as features.

#### Affective Ratings of Emotions

**Figure 1:**
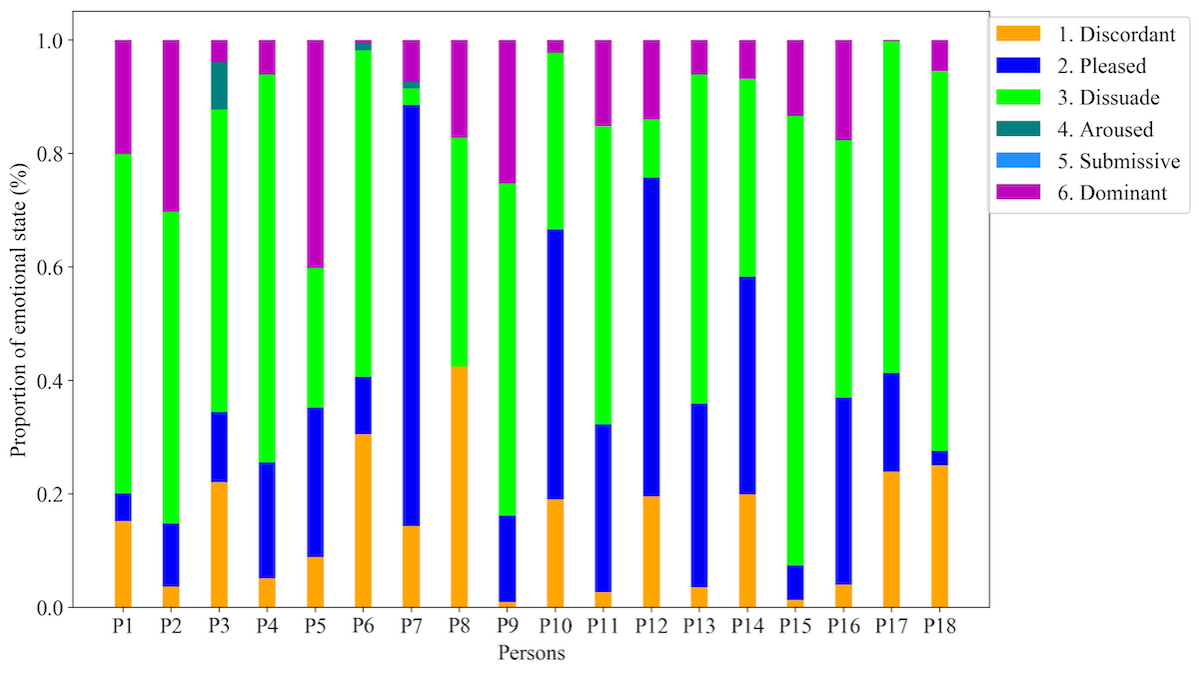
Proportion (%) of 6 emotional states per person. Pleased and dissuaded are the most frequent, whereas submissive and aroused are the least frequent emotional states among the 18 persons in our data set.

In this study, we used the Affective Norms for English Words (ANEW) [26] to map the 49 discrete emotions to the PAD model. ANEW was developed by the Center for the Study of Emotion and Attention to provide standardized materials to researchers studying emotion and attention. The latest ANEW database [27] contains affective meanings of nearly 14,000 English lemmas rated by a larger cohort of 1827 participants with a wide range of diversities, including age, occupation, and educational differences. We used the latest database to map the 49 emotions to the 3 ratings of pleasure (*p*), arousal (*a*), and dominance (*d*). Therefore, each linguistic emotion label was converted into 3 continuous values on a scale of 1 to 9, where 1 and 9 indicate the lowest and highest intensity, respectively, in the corresponding PAD dimension. A list of the 49 emotions used in this study with their corresponding PAD values is included in Multimedia Appendix 1 [22,23,28]. We scaled the emotional ratings (*R_s_*) in the range of –4 to +4 by subtracting 5. Then, we considered 6 states of emotions on the basis of the intensity (sign) in the 3 dimensions: discordant, pleased, dissuaded, aroused, submissive, and dominant. We calculated the prevailing emotional state at any point of time for a person by considering the absolute maximum value of (*R_s_*) and its sign. The absolute maximum value indicates the dimension, whereas the sign represents the direction. Therefore, the emotional state at any point represents which of the 3 dimensions is prevailing and in what direction. For example, the emotional rate (*R_s_*) of *happy* is 8.47, 6.05, and 7.21 *for p, a,* and *d*, respectively. The corresponding scaled values will be 3.47, 1.05, and 2.21, respectively. Here, the 3 values are positive, and the prevailing emotional state is (+*p)* pleased. Therefore, this emotional state will be assigned to the class *pleased.* Similarly, *angry* (*p*=2.53, *a*=6.2, and *d=*4.11) will be scaled to –2.47, 1.2, and –0.89, respectively, with a maximum absolute value of 2.47 and in the negative direction. Hence, *angry* will be assigned to the class *discordant*. There was 1 case (the emotion *interested*)*, where p* and *a* were equal*.* In this case, we had 2 dominant dimensions, and we chose the first positive value (in the order of *PAD*), *p*, to represent the dominant emotion. Although this is a limitation, there was only 1 emotion *interested* that had 2 equal values, and there were few cases with this emotion in the data set compared with the other 48 emotions. Although we considered 6 categories of emotional states, not all classes were present in every person’s data. Depending on the person, 1 or 2 emotional states were absent in the data set. Figure 1 shows the different emotional states present in each person’s data.

#### Feature Engineering

Initially, we merged all features from sensors, location data, and self-reported contextual information. Sensor measurements were recorded for 20 seconds every minute, and the data collection period varied from 3 to 9 days for each person. The number of samples for each person varied from 1164 to 6263. The data set contains a mixture of binary and continuous variables. We also engineered 7 additional temporal and spatial features from timestamps and location data. Overall, the features can be categorized as follows.

##### Motion

We considered 138 features calculated from the raw measurements from 3 smartphone sensors (an accelerometer, a gyroscope, and a magnetometer) and 2 smartwatch sensors (an accelerometer and a compass). These are continuous variables.

##### Audio

We considered 28 naive features calculated as the averages and standard deviations of the 13 Mel Frequency Cepstral Coefficients from the approximately 20-second recording window and the overall power of the audio.

##### Location

We considered 17 location features measured from the relative locations and movement variability in every minute of persons. We also feature engineered 3 more location features: *cl_latitude*, *cl_longitude*, and *geo_dist*. We clustered the neighboring latitudes and longitudes using geohash [29]. Geohash is a geocoding system invented by Niemeyer [29] that enables the grouping of neighboring points in a rectangular cell defined by a precision value. We used a precision value of 8 to cluster the neighboring latitude and longitude within 38.2 m × 19.1 m. The rectangular box worked as a bounding box for all neighboring spatial points falling into this area. After geohashing, the geocodes were decoded back to clustered latitude (*cl_latitude*) and longitude (*cl_longitude*) values. We calculated the *geo_dist* feature as the Haversian distance traveled by the person since the previous time stamp.

##### Phone State

We considered 28 binary features that indicate the sensed state of the phone, such as app states, battery plugged, battery states, ringer mode, on the phone, Wi-Fi status, screen brightness, and battery level.

##### Environmental

The ExtraSensory data set also contains 6 environmental variables such as light, pressure, humidity, and temperature. All of these continuous variables were included in our primary feature list. However, there were many missing values for these features because not all phones had all the sensors.

##### Temporal

We engineered 5 variables from the recorded time stamps to explore the temporal pattern of emotional states and transitions: *minute of the hour*, *minute of the day*, *hour of the day*, *day of week*, and *time difference in minutes*. As the data set was very sparse, we calculated the *time difference in minutes* variable to measure how many minutes elapsed since the last record. The remaining 4 variables were categorical variables.

##### Contextual

We also considered 51 binary contextual labels such as indoor, outdoor, eating, and in a car, which were self-reported by the subjects at various intervals. We assumed that this self-reported information was correct in all cases to focus on automatically recognizing the dominant emotion without dealing with noisy estimates of the context. Although the latest ExtraSensory app [28] is capable of recognizing contextual information passively on the basis of raw sensor data, the data set used in this study did not include the output from this new feature.

A complete list of features is included in Multimedia Appendix 1.

#### Data Resampling, Cleaning, and Imputation

Our study aims to detect emotional transitions and states in small time intervals. Therefore, we resampled all data to a frequency of every 5 min. In the original data, the number of samples in 5-min intervals varied from 0 to 5. During resampling, we calculated the average of all continuous variables, the summation of all binary variables, and the maximum of all ordinal variables for all samples within the 5-min interval. This allowed us to have an evenly spaced sampling frequency over time and reduced missing data. All missing values were replaced by a large negative number to indicate missingness. Features were standardized by removing the mean and scaling to unit variance. This was done on the basis of the training sets.

#### Ethics Approval

As ExtraSensory is a public data set, research ethics approval was waived.

### Emotional Transition and State Detection

#### Feature Handling

For emotional transition detection, we considered the changes in features from the previous window. Therefore, the feature set *T_t,k_* at any time *t* was calculated as follows:

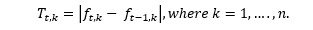



In equation 1, the total number of features is n, *f_t,k_* represents the value of the *k*th feature at the *t*th window, and *f_t_*_−1,_*_k_* represents the value of the *k*th feature at the *(t-1)*th window. The intuition was to feed the ML models with the changes in information to find any pattern associated with changes in the captured data. The original form of the resampled features was used for emotional state detection.

Next, we analyzed the features to select a smaller set of important features. We selected the k best features for each model by applying the SelectKBest feature selection function from the feature_selection package provided by sklearn, where the following values were experimented for k: 50, 70, 90, and 110. This feature selection process was applied independently for emotional transition and state detection and for general and personalized models. This resulted in a different number of features for each model. Additionally, columns with more than 30% missing data were removed. Location data were removed from the general models to make the models as generalizable as possible.

#### Machine Learning Models

We developed general models for all individuals as well as personalized models for each person to explore the impact of interpersonal variability on the performance of emotional transition and state detection.

**Figure 2:**
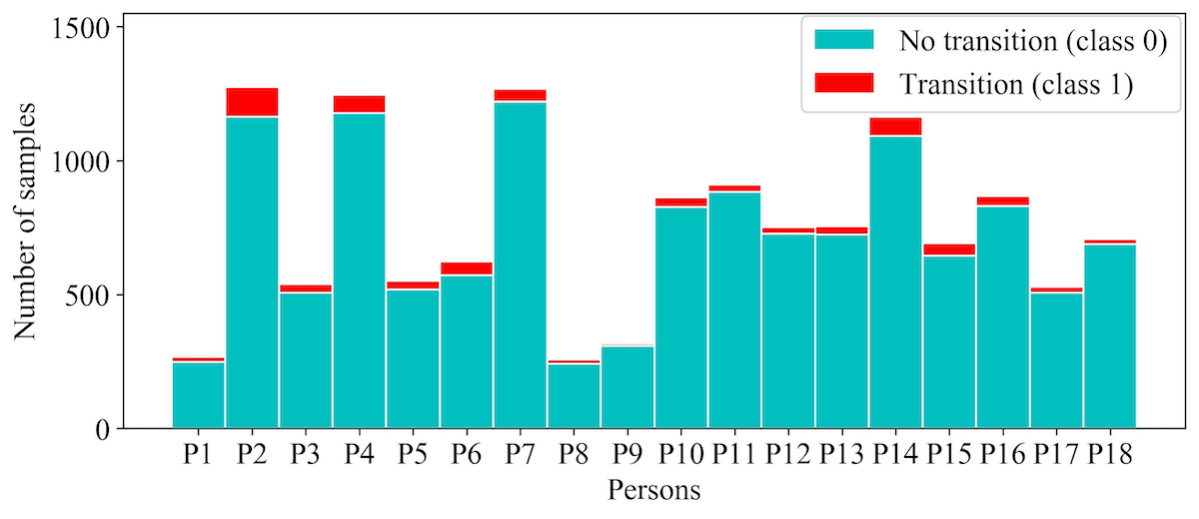
A stacked bar chart of the distribution of the 2 classes (transition and no transition) per person.

We used 5 supervised ML algorithms: logistic regression (LR), random forest (RF), XGBoost (XGB), CatBoost (CB), and multilayer perceptron. Emotional transition detection is a binary classification problem, where 0 and 1 denote no change and change in emotional state, respectively, over the last 5-min window. Owing to the sparsity of the data, the target variable was overly imbalanced. Figure 2 shows the class imbalance of emotional state transitions of the 18 persons. Hence, we also applied 2 imbalance handling techniques that we explain in the *Imbalance Handling* section below.

Emotional state detection is a multiclass classification problem, where we intended to classify the prevailing emotional state of a person at a given time into one of the following 6 classes: 1, discordant; 2, pleased; 3, dissuaded; 4, aroused; 5, submissive; and 6, dominant. However, as shown in Figure 1, the emotional state classes were also imbalanced. We removed any class from a person’s data having less than or equal to 6 samples. As a result, the number of classes varied from 3 to 5 for each person.

Hyperparameters for each model were determined using a stratified cross-validated grid search over a parameter grid. In the general models, we used a six-fold, leave-3-people-out cross-validation, where for each fold, the models were trained on 15 individuals’ data and tested on the remaining 3 data points. Hyperparameters were tuned by optimizing the *F*_1_ score. The tuned hyperparameters are listed in Multimedia Appendix 1. For the personalized models, a five-fold, stratified cross-validation was used to fine-tune the hyperparameters and select the best-performing models. The total number of samples per person varied from 257 to 1268. We measured 7 performance metrics to evaluate the classification performance: accuracy, balanced accuracy, precision, recall, *F*_1_ score, specificity, and area under the receiver operating characteristic (AUROC) curve. For emotional state detection, the macro precision, recall, *F*_1_ score, specificity, and AUROC curve were measured to emphasize the detection performance for the minority classes.

#### Imbalance Handling

In this study, Synthetic Minority Over-sampling Technique (SMOTE) and Support Vector Machines Synthetic Minority Over-sampling Technique (SVMSMOTE) [30] were applied to mitigate class imbalance. These are oversampling methods that create synthetic data of the minority classes to decrease the imbalance. All imbalance handling techniques were applied only on training data to avoid data leakage between training and test sets.

#### Feature Analysis

We analyzed the importance of the 7 categories of features used in this study for both emotional transition and state detection. The detection performance of emotional transition and state varied for different persons, which we categorized as best, average, and worst performances. We used the output of the XGB classifier for the feature importance analysis to explore the best-performing features.

### Software

This study was conducted in Python 3 with the following packages: Scikit-Learn (0.22), CB, XGB, and SHapley Additive exPlanation (SHAP). Python codes are publicly available on GitHub [31].

## Results

A summary of the data set containing the number of days and the percentage of missing data is presented in Table 1. Table 1 shows that the average amount of missing values in the data set was approximately 63%, where the range varied from 38.66% to 88.8% for different persons.

**Table 1:** Summary of the 18 persons’ (P1-P18) data used in this study.

Person	Number of days	Data (5-min window)	
		Windows with complete data, n	Missing data^a^ (%)
P1	3	268	68.9
P2	8	1275	44.62
P3	7	539	73.21
P4	8	1245	45.92
P5	8	551	76.04
P6	8	623	72.92
P7	8	1268	44.92
P8	8	257	88.80
P9	7	317	84.23
P10	8	862	62.54
P11	8	911	60.42
P12	8	752	67.32
P13	9	755	70.83
P14	7	1164	42.21
P15	8	692	69.92
P16	7	868	56.89
P17	3	529	38.7
P18	7	707	64.88
Average (SD)	7.22 (1.63)	754.61 (328.06)	62.96 (14.71)

^a^Missing value is the percentage of missing windows (time slots).

### Emotional Transition or State Detection Results

In terms of general models for emotional transition detection, the best-performing classifiers were LR, XGB, and CB. Table 2 shows the results of these classifiers. As shown in Table 2, the best average AUROC curve of 90.5% was obtained by LR, followed by XGB and CB with 89.72% and 89.24%, respectively. The table also shows that using imbalance handling improved average recall only but did not improve the results in general. In general, SVMSMOTE produced better results in terms of imbalance handling than SMOTE for most models. Complete results including the results using SMOTE are shown in the Multimedia Appendix 1.

**Table 2:** Results of the general models for emotional transition detection on the basis of a six-fold, leave-3-people-out cross-validation.

Characteristics	Machine learning models^a,b^ (without imbalance handling)			Imbalance handling using Support Vector Machines Synthetic Minority Over-sampling Technique		
	LR^c^	XGB^d^	CB^e^	LR	XGB	CB
Accuracy, % (SD)	*94.77 (1.64)*	94.50 (1.54)	94.60 (1.58)	91.16 (1.24)	5.33 (1.46)	41.31 (33.75)
Balanced accuracy, % (SD)	60.59 (4.26)	*66.92 (5.41)*	65.02 (5.47)	80.76 (4.68)	50.09 (0.18)	60.56 (10.45)
Precision (macro), % (SD)	50.08 (10.55)	*51.77 (16.98)*	51.01 (12.13)	32.59 (5.47)	5.17 (1.58)	9.37 (4.73)
Recall (macro), % (SD)	22.32 (8.49)	36.03 (11.70)	31.98 (12.32)	69.05 (9.46)	*100.00 (0.00)*	82.27 (20.45)
Specificity (macro), % (SD)	*98.85 (0.44)*	97.81 (1.29)	98.05 (1.53)	92.48 (0.99)	0.17 (0.36)	38.84 (36.35)
AUROC^f^ (macro), % (SD)	*90.50 (3.01)*	89.72 (2.51)	89.24 (2.51)	90.26 (3.20)	60.49 (14.11)	74.77 (9.75)
*F*_1_ (macro), % (SD)	29.89 (9.51)	38.85 (7.83)	36.46 (6.72)	*43.63 (5.05)*	9.80 (2.83)	15.78 (6.53)

^a^Average (SD) across six-fold.

^b^The highest value of each metric is italicized.

^c^LR: logistic regression.

^d^XGB: XGBoost.

^e^CB: CatBoost.

^f^AUROC: area under the receiver operating characteristic.

Table 3 reports the performance measures for the general models for emotional state detection. As shown in Table 3, the best results were obtained from LR, CB, and RF. In particular, LR achieved the best average AUROC curve of 60.23%. Adding imbalance handling slightly improved some of the metrics such as specificity and balanced accuracy. The full results from all models and imbalance handling techniques can be found in Multimedia Appendix 1.

**Table 3:** Results of the general models for emotional state detection on the basis of a six-fold, leave-3-people-out cross-validation.

Characteristics	Machine learning models^a,b^ (without imbalance handling)			Imbalance handling using Support Vector Machines Synthetic Minority Over-sampling Technique		
	LR^c^	CB^d^	RF^e^	LR	CB	RF
Accuracy, % (SD)	40.60 (9.50)	*44.10 (13.93)*	44.04 (14.42)	32.61 (2.65)	39.52 (13.24)	38.99 (8.71)
Balanced accuracy, % (SD)	22.83 (2.64)	24.32 (3.23)	21.84 (2.03)	*30.66 (8.82)*	23.32 (2.91)	23.27 (2.43)
Precision (macro), % (SD)	*34.25 (5.26)*	29.52 (11.39)	20.36 (7.50)	26.48 (2.32)	27.18 (10.23)	25.33 (2.20)
Recall (macro), % (SD)	*38.86 (6.68)*	33.29 (8.57)	29.40 (3.87)	28.27 (7.76)	27.81 (5.77)	25.94 (2.08)
Specificity (macro), % (SD)	63.37 (12.40)	72.40 (3.62)	71.23 (4.73)	*82.93 (0.76)*	75.36 (4.66)	76.93 (2.63)
AUROC^f^ (macro), % (SD)	60.23 (8.15)	58.58 (6.97)	55.21 (4.31)	*60.55 (3.41)*	56.83 (6.14)	55.43 (4.37)
*F*_1_ (macro), % (SD)	*30.60 (5.83)*	25.45 (10.72)	19.20 (5.19)	23.04 (2.82)	21.20 (6.95)	21.81 (3.24)

^a^Average (SD) across six-fold (average value for each metric).

^b^The highest value of each metric is italicized.

^c^LR: logistic regression.

^d^CB: CatBoost.

^e^RF: random forest.

^f^AUROC: area under the receiver operating characteristic.

For the personalized emotional transition detection models, all models performed quite well in general, and it was not possible to pinpoint 1 single best ML model and imbalance handling technique for all the 18 persons. Table 4 reports the performance measures obtained from RF, XGB, and CB. The standard deviations of the measures indicate large variabilities across 18 persons. The best measures highlighted in Table 4 demonstrate the variabilities of the measures across different ML models. As shown in Table 4, the best average AUROC curve of 88.01% was obtained by RF without imbalance handling, whereas SVMSMOTE helped improve recall in general (especially in XGB) and produced the best average AUROC curve of 88.7% with CB. The detailed results of all classifiers and imbalance handling techniques are listed in the Multimedia Appendix 1.

**Table 4:** Results of the personalized models for emotional transition detection on the basis of the 5-fold, stratified cross-validation.

Characteristics	Machine learning models^a,b^ (without imbalance handling)			Imbalance handling using Support Vector Machines Synthetic Minority Over-sampling Technique		
	RF^c^	XGB^d^	CB^e^	RF	XGB	CB
Accuracy, % (SD)	93.49 (4.73)	*94.82 (2.55)*	94.29 (3.17)	90.31 (7.17)	89.54 (6.06)	92.34 (5.01)
Balanced accuracy, % (SD)	66.88 (10.08)	65.91 (7.93)	66.89 (8.97)	66.80 (5.93)	*75.86 (8.52)*	70.28 (8.06)
Precision (macro), % (SD)	49.97 (21.23)	*52.34 (17.22)*	48.67 (18.09)	34.29 (17.13)	35.69 (13.32)	42.37 (14.18)
Recall (macro), % (SD)	37.12 (20.78)	33.71 (15.69)	36.45 (18.10)	40.77 (13.49)	*60.76 (16.37)*	45.82 (15.17)
Specificity (macro), % (SD)	96.65 (4.91)	*98.12 (1.42)*	97.34 (2.33)	92.82 (7.63)	90.97 (6.03)	94.75 (4.53)
AUROC^f^ (macro), % (SD)	88.01 (5.67)	87.84 (6.81)	87.62 (7.04)	85.08 (7.20)	87.74 (6.63)	*88.73 (6.24)*
*F*_1_ (macro), % (SD)	36.40 (17.87)	38.00 (15.92)	38.34 (17.07)	32.92 (12.48)	*41.85 (13.46)*	40.44 (13.88)

^a^Average (SD) across 18 persons.

^b^The highest value of each metric is italicized.

^c^RF: random forest.

^d^XGB: XGBoost.

^e^CB: CatBoost.

^f^AUROC: area under the receiver operating characteristic.

Compared with the performance of the general models for the emotional state detection task, the performance of the personalized models was substantially better. Table 5 reports the performance measures obtained from CB, XGB, and RF. As shown in Table 5, the best average AUROC curve of 96.33% was obtained by CB followed by XGB and then RF. Applying imbalance handling techniques slightly improved the balanced accuracy, recall, and specificity. While all classes were maintained in the general models, the number of classes varied between 4 and 5 across the personalized models. Complete results including those from SMOTE are shown in the Multimedia Appendix 1.

**Table 5:** Results of the personalized models for emotional state detection on the basis of a five-fold, stratified cross-validation.

Characteristics	Machine learning models^a,b^ (without imbalance handling)			Imbalance handling using Support Vector Machines Synthetic Minority Over-sampling Technique		
	CB^c^	XGB^d^	RF^e^	CB	XGB	RF
Accuracy, % (SD)	*86.53 (8.08)*	82.73 (7.18)	80.92 (10.69)	85.27 (9.64)	81.54 (8.90)	78.21 (10.78)
Balanced accuracy, % (SD)	74.92 (13.55)	69.51 (12.18)	67.85 (14.10)	*77.73 (13.34)*	74.50 (12.03)	70.72 (13.92)
Precision (macro), % (SD)	*87.03 (9.37)*	82.49 (7.32)	82.11 (8.67)	84.44 (9.83)	77.71 (11.09)	75.94 (12.02)
Recall (macro), % (SD)	77.88 (11.35)	74.54 (7.70)	71.96 (10.87)	*80.00 (10.74)*	77.23 (10.36)	73.30 (11.91)
Specificity (macro), % (SD)	92.92 (4.80)	90.48 (5.64)	89.83 (6.58)	*93.15 (4.84)*	91.96 (4.71)	90.19 (5.67)
AUROC^f^ (macro), % (SD)	*96.33 (3.26)*	94.81 (2.96)	93.74 (5.62)	96.26 (3.77)	94.51 (3.39)	92.93 (5.27)
*F*_1_ (macro), % (SD)	*79.48 (11.38)*	74.87 (8.45)	72.46 (12.07)	79.47 (12.00)	74.51 (12.05)	70.73 (13.34)

^a^Average (SD) across 18 persons.

^b^The highest value of each metric is italicized.

^c^CB: CatBoost.

^d^XGB: XGBoost.

^e^RF: random forest.

^f^AUROC: area under the receiver operating characteristic.

### Feature Analysis Results

**Figure 3:**
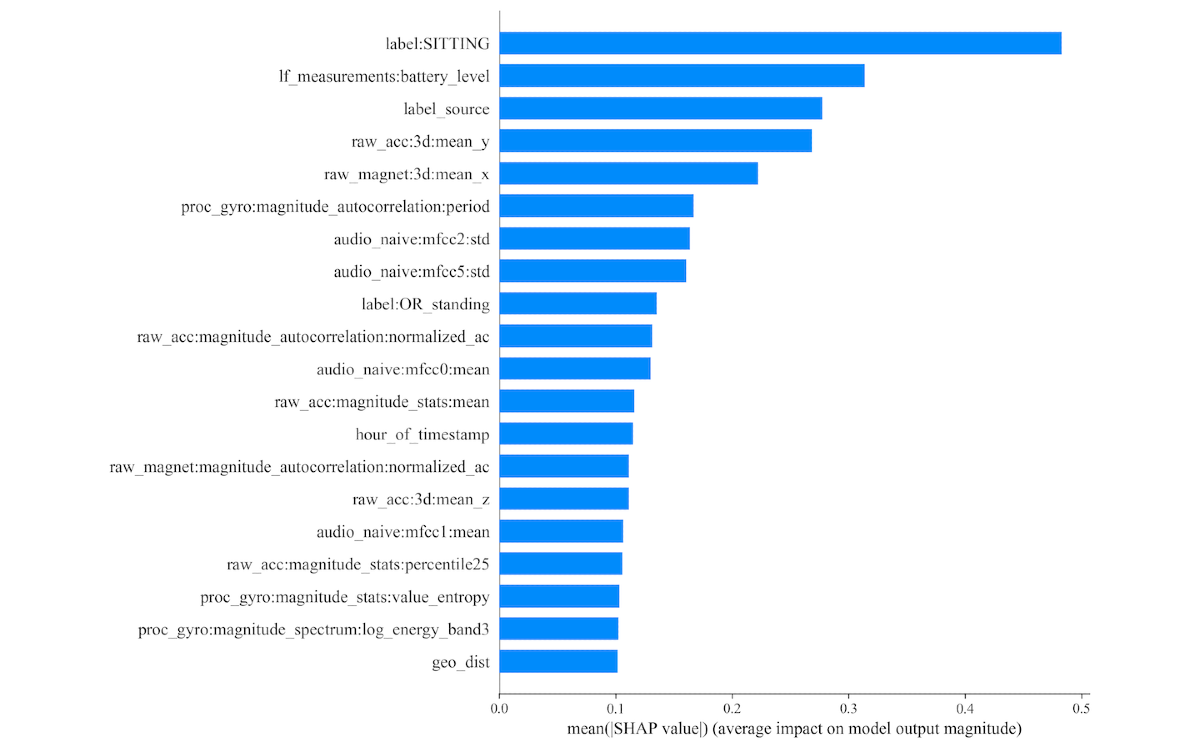
Feature importance for emotional transition detection of the general model obtained using XGBoost and shapley additive explanation. The figure represents the contribution of the corresponding feature to detect whether there is a transition.

**Figure 4:**
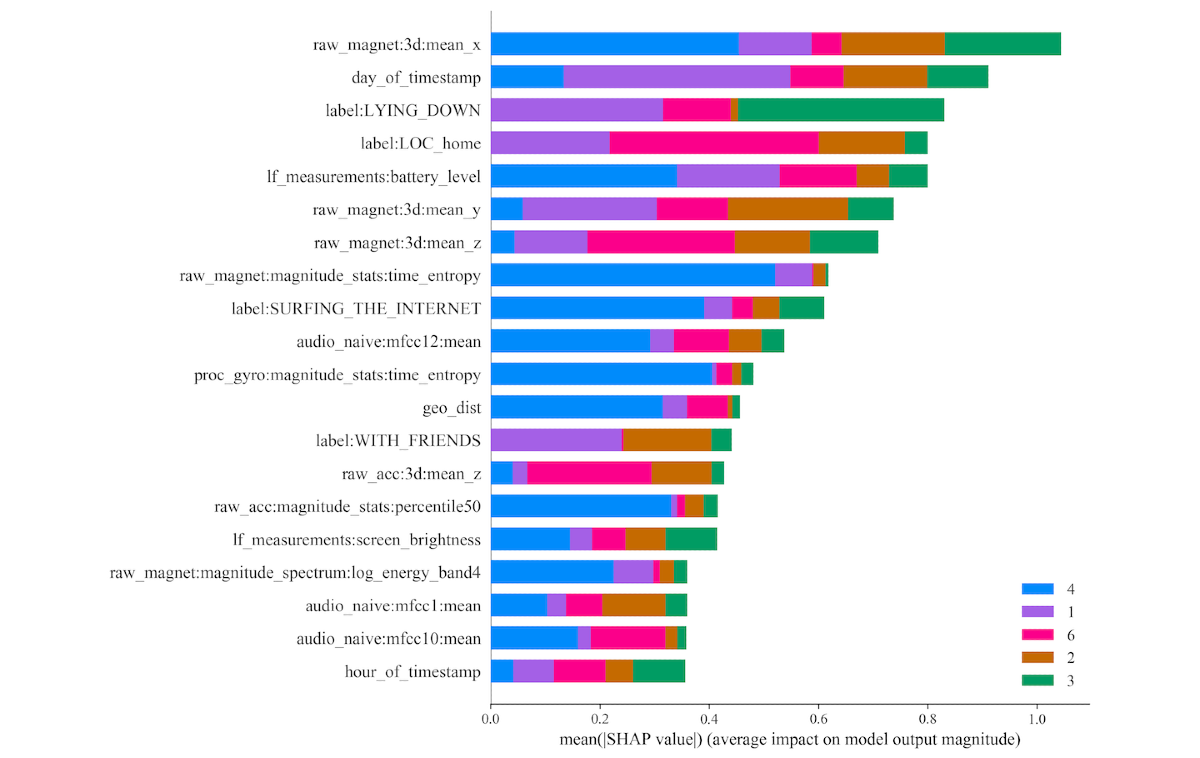
Feature importance for emotional state detection of the general model obtained using XGBoost and shapley additive explanation. The color-coded portions represent the contribution of the corresponding feature to detect different states (classes) of emotions: 1, discordant; 2, pleased; 3, dissuaded; 4, aroused; 5, submissive; and 6, dominant. SHAP: SHapley Additive exPlanation.

Using the output of the XGB classifier for feature importance analysis, we explored the best-performing features. Figures 3 and 4 show the 20 most important features of the general model using XGB and SHAP [32] for emotional transition and state detection, respectively. The contextual information (prefix *label*) ranked higher among the 7 categories of features. Contextual information features appear among the top 4 features for both emotional transition and state detection. Other important features included motion, phone state, and temporal for both emotional transition and state detection.

**Figure 5:**
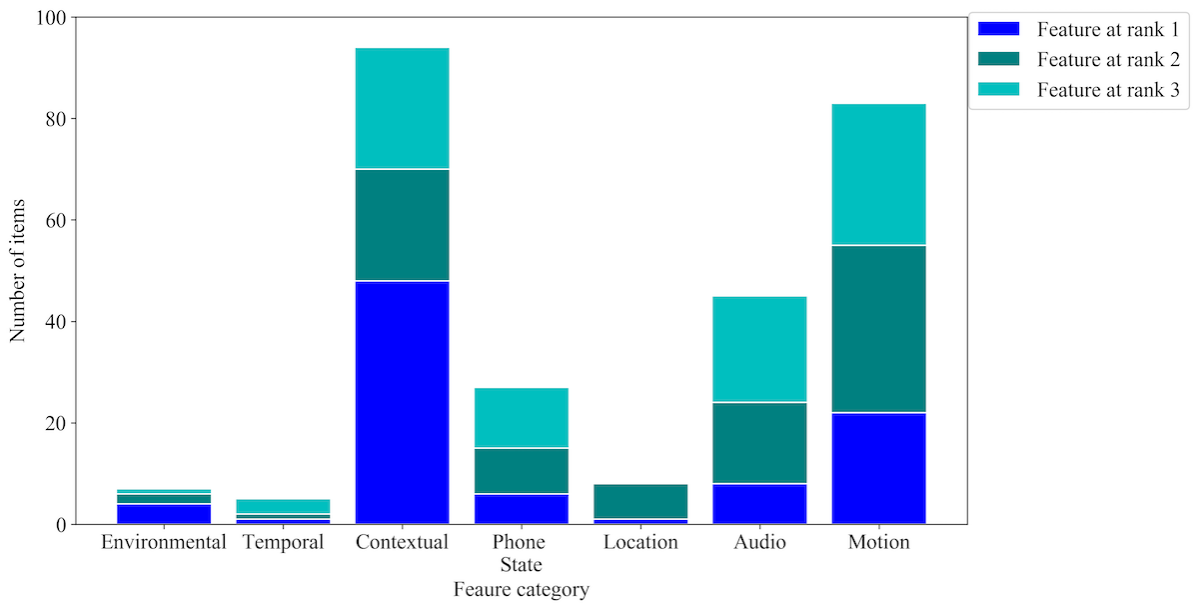
Importance of feature categories for emotional transition detection. The color-coded portions of each category represent their contributions to the top 3 ranks of features of 18 persons.

**Figure 6:**
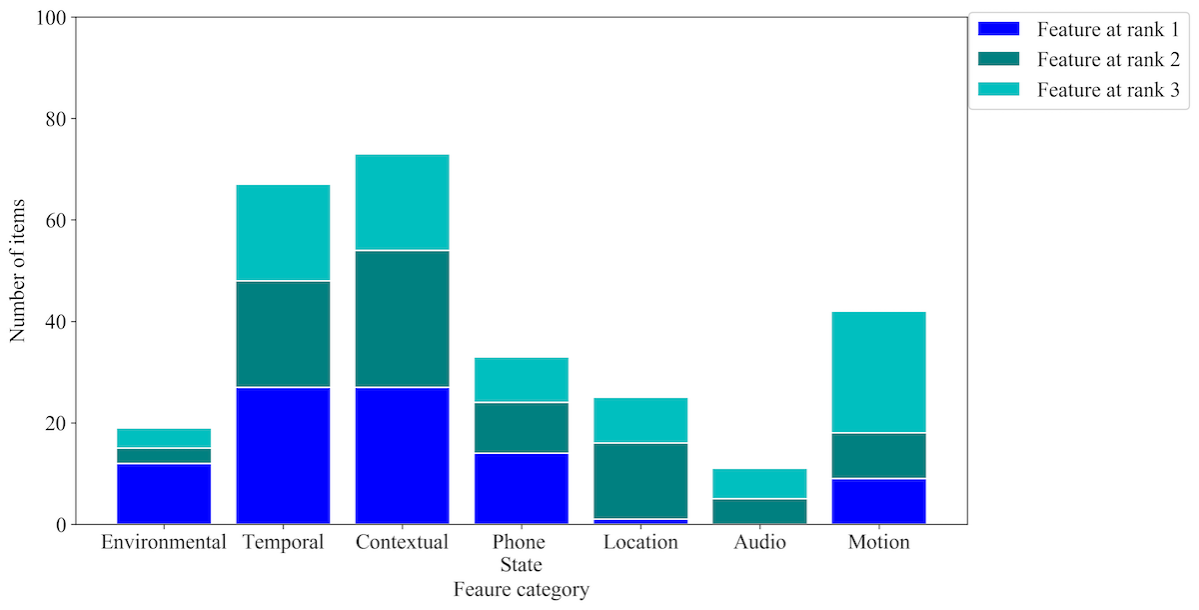
Importance of feature categories for emotional state detection. The color-coded portions of each category represent their contributions to the top 3 ranks of features of 18 persons.

We also explored the influential feature categories by considering the 3 most important features across all 18 persons. Figures 5 and 6 demonstrate the importance of the 7 feature categories for emotional transition and state detection, respectively. This was done manually; each category is a bin of a set of features as described in the *Feature Engineering* section. Instead of the feature itself, we considered which bins the top-3 features belong to. This was done over folds for each person. For calculation, we considered a3 empty matrix, where 7 is for each category and 3 is for the top 3 ranks. Then we incremented the counter of the corresponding category and ranked the top 3 features of each fold for each person. For example, in Figure 3 (although for a general model but for the sake of explanation), the top 3 features are *label: SITTING*, *If_measurement:battery_level*, and *label: Source*, where the first and third features belong to the contextual category and the second feature falls under the phone state category. This will increase the first and third rank counters of the contextual category and the second rank counter of the phone state category. This was done based on the XGB classifier and SHAP.

Figure 5 shows that the most important feature categories for emotional transition detection are contextual, motion, and audio signals, whereas the least important categories are temporal and environmental features. In contrast, as shown in Figure 6, the most important feature categories for emotional state detection are contextual and temporal, whereas the least important category is audio signals. Unlike emotional transition, emotional state was more influenced by environmental and location features. This also explains why the personalized emotional state detection models performed better than the general models where the location data were ignored. However, contextual data played the most important role in both emotional transition and state detection.

## Discussion

The 4 major findings of this exploratory study are as follows:

- Emotional transitions in small intervals are detectable from data captured via smartphones and smartwatches using ML techniques. We observed that the contextual data, sensed phone states, and motion-related signals are the most influential features for emotional transition detection.
- The prevailing emotional states and the direction in the 3 dimensions of the PAD model are detectable by applying ML algorithms to information captured by smartphones and smartwatches. The contextual and temporal data play important roles in the detection of emotional states in small time intervals.
- We observed a wide range of interpersonal variations in terms of the detectability of emotional transitions or states. In terms of the personalized models, no single ML model performed the best across all 18 persons.
- Personalized models can better detect emotional states than general models. We believe that a given individual’s data contributes most to detecting their own emotional state rather than using data from other individuals.

This study shows the feasibility of detecting persons’ emotional states and transitions passively by training ML models on the daily data sensed via smartphones and smartwatches. In practice, these findings will help in reducing self-reports, enabling seamless tracking of daily emotions. For example, a person can be prompted to verify his or her emotional states only if the smartphone app senses a transition in the emotional state of the person. In addition, it can provide the capability of tracing the influential contextual variables that trigger the transition, which can be adjusted by the person for better self-management and well-being. Our investigation showed that self-reported contextual information played the most important role in both emotional transition and state detection. Although the contextual data are self-reported in this data set, latest research shows that contextual data can be labeled from sensed data by smartphone apps automatically without human intervention [28].

The emotional transition detection results obtained in this study are promising but not as good as the emotional state prediction performance. One reason for this could be the highly imbalanced classes, which can be resolved largely by acquiring data for a longer period of time (we had a noticeable improvement in the general models). Moreover, performance can be improved by combining contextual information with other dominant factors of emotions such as personality traits, social communication (eg, incoming-outgoing phone calls, duration, text messages, social media usage), and physiological signals (eg, heart rates, skin conductance) captured via smartphones and smartwatches.

Unlike emotional transition detection, the performance of general models for emotional state detection was poor. Apart from being a harder task (six-class classification), we believe this is also due to the high variability across different individuals. Many state-of-the-art studies stressed the need for further research on interperson variations in affect, mood, and mental health [11,33]. One of the aims of our research was to address this identified gap by exploring subjective variabilities in emotional state and transition detection. Therefore, in addition to the general models, we built personalized models. In these personalized models, each model was trained and tested on each person’s data to explore the impact of interpersonal variability on the performance of emotional transition and state detection. The results of the personalized and generic models of this study bolster the need to consider subjective variability while building ML models for emotional state and change detection.

We also observed that simpler models, such as LR, performed better for emotional transition detection and complex models, outperformed during emotional state detection. One reason for this could be the consideration of changes in features between 2 consecutive windows that made the data set sparser (containing many 0s) and smaller for emotional transition detection. Although the personalized models performed better than the general models, especially in the emotional state detection task, the general models can be used as baseline models, which can subsequently be personalized for each person.

Although research has suggested the inclusion of dominance for a better understanding of emotional states [21], it was ignored in the existing works on emotional state and mood recognitions. Our study showed that the prevailing emotional state and its direction in all the 3 dimensions can be detected using ML models on contextual information and data sensed via mobile phones and wearable devices. It can provide data-driven insights on which of the 3 dimensions of emotion prevailed for the person when, where, and in what direction, eventually leading the person toward effective lifestyle changes and better self-management.

**Figure 7:**
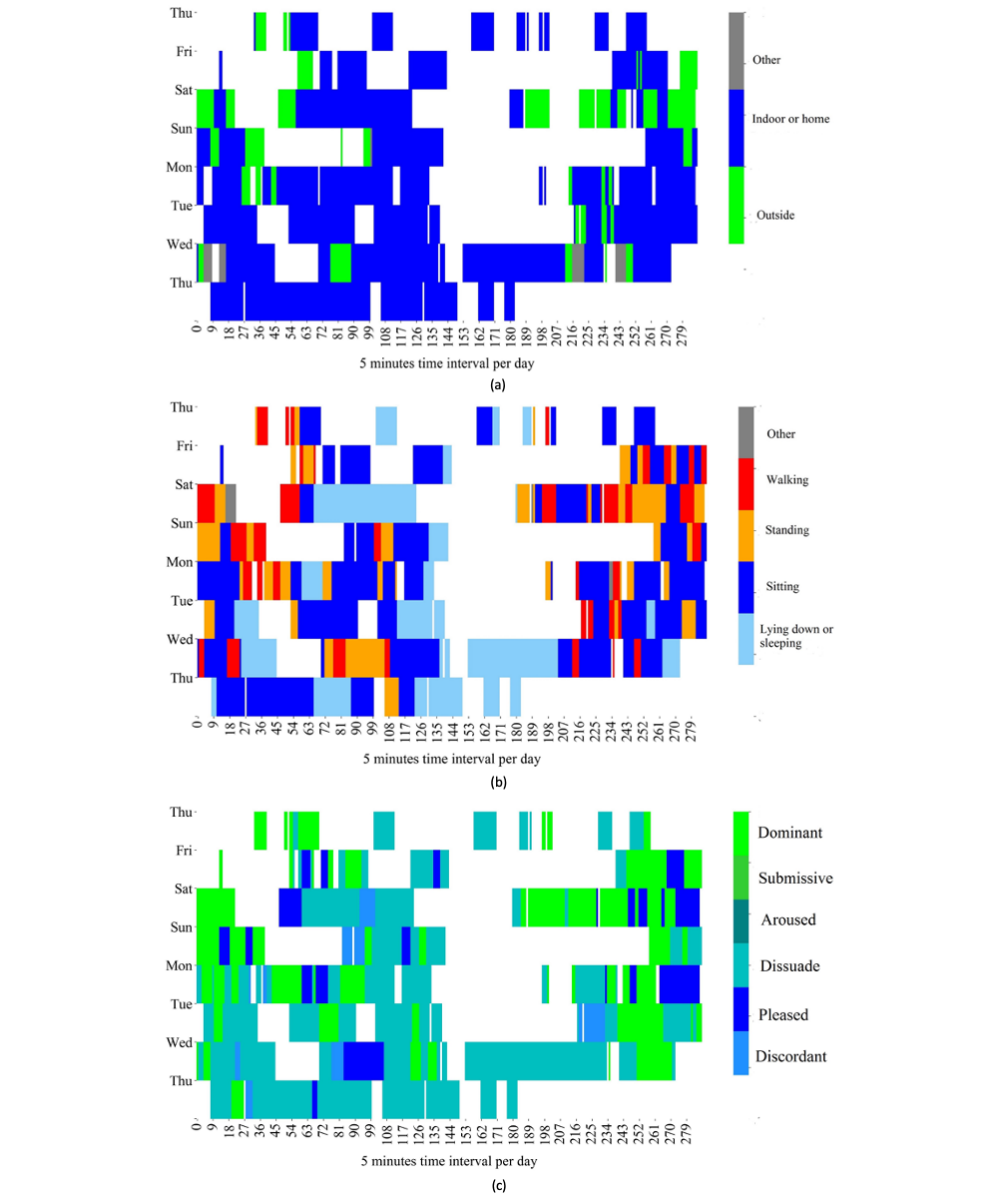
Daily life versus emotional states of person 2 (worst case). The x-axis plots 288 windows per day, and the y-axis plots the number of days in the data collection period of person 2. The color-coded regions represent (a) spatial contexts, (b) activities, and (c) emotional states in each window over the period of data collection (8 days). The white regions represent missing data.

**Figure 8:**
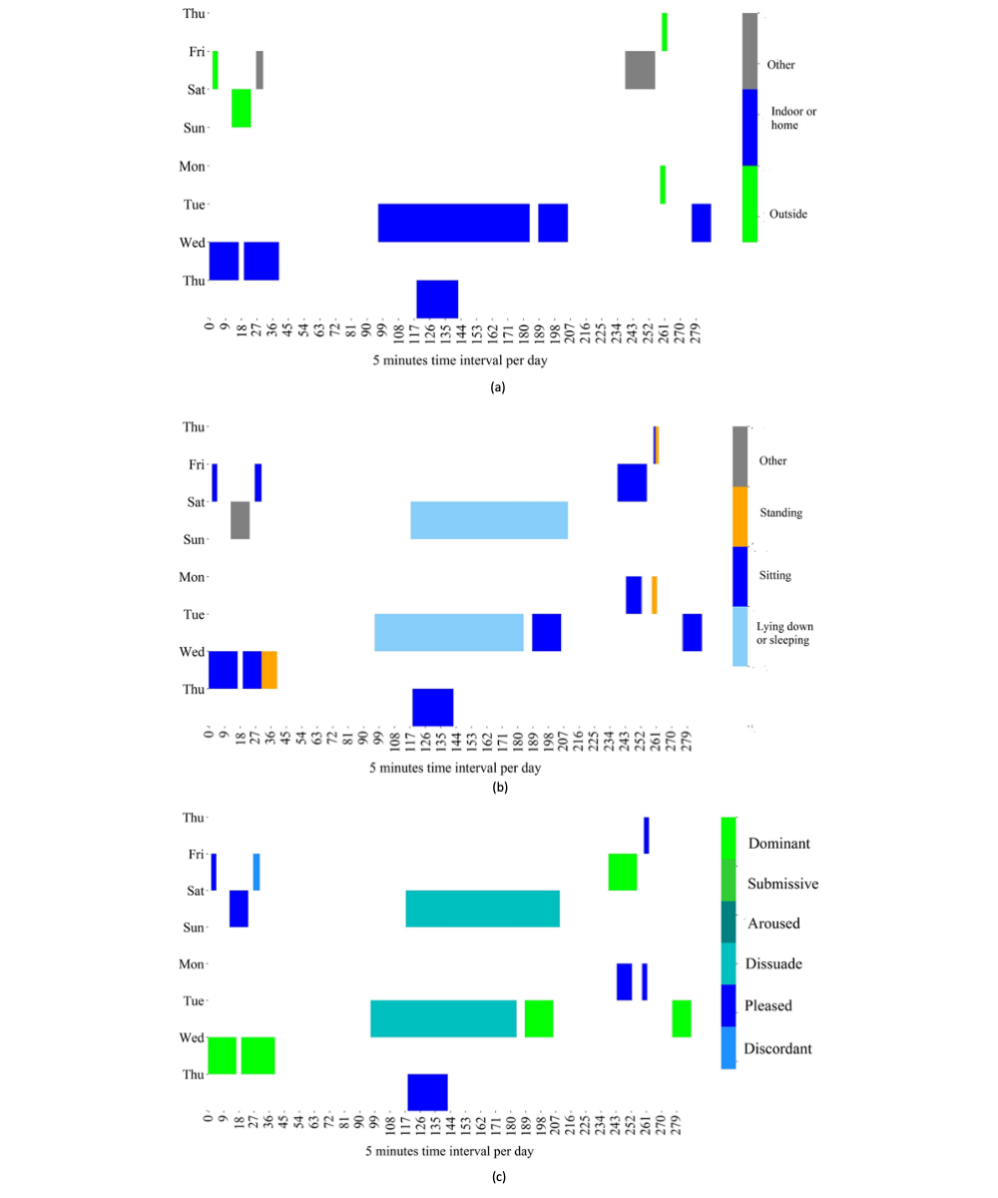
Daily life versus emotional states of person 9 (best case). The x-axis plots 288 windows per day, and the y-axis plots the number of days in the data collection period of person 9. The color-coded regions represent (a) spatial contexts, (b) activities, and (c) emotional states in each window over the period of data collection (8 days). The white regions represent missing data.

**Figure 9:**
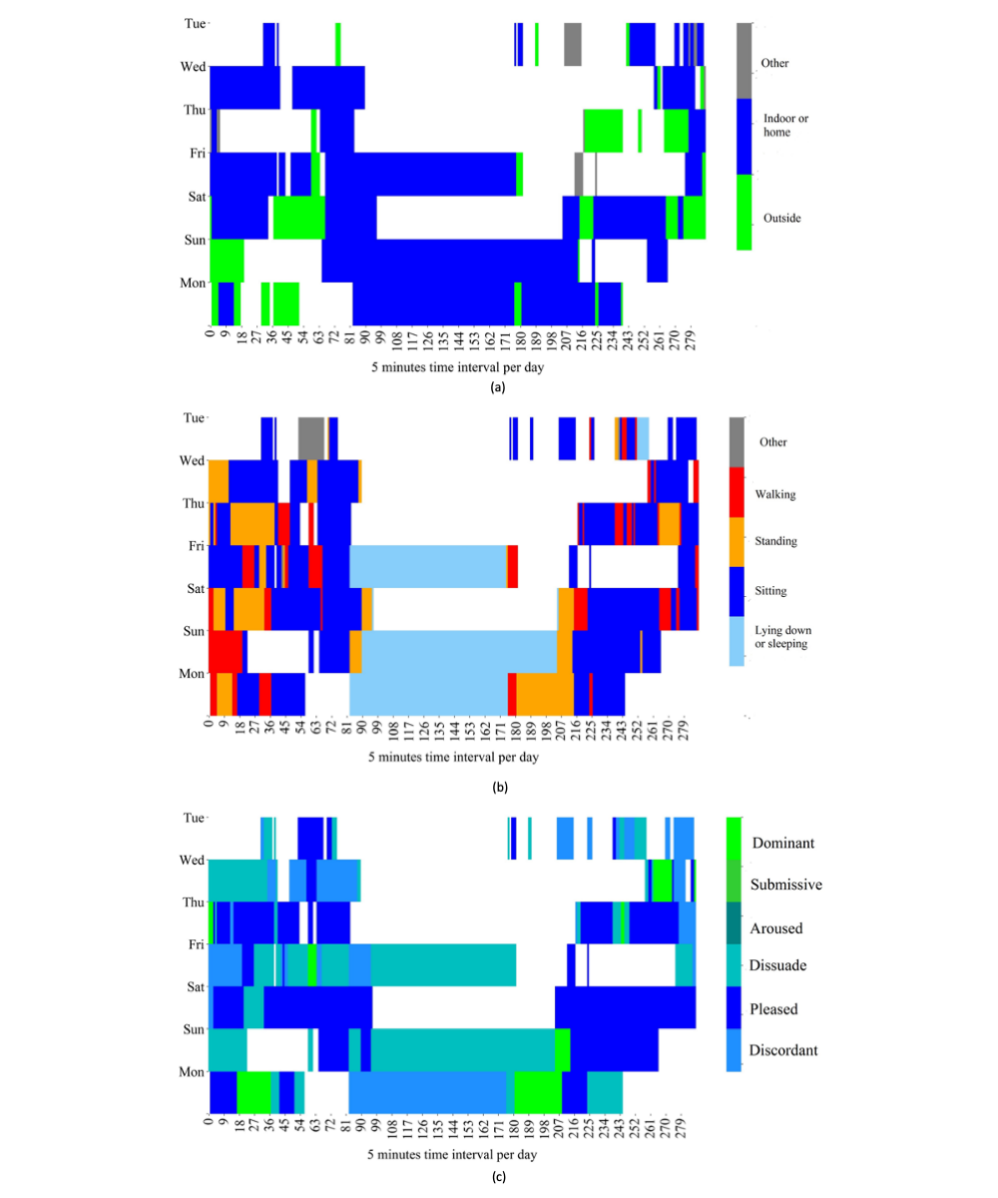
Daily life versus emotional states of person 14 (average case). The x-axis plots 288 windows per day, and the y-axis plots the number of days in the data collection period of person 9. The color-coded regions represent (a) spatial contexts, (b) activities, and (c) emotional states in each window over the period of data collection (7 days). The white regions represent missing data.

Our study shows that a large number of interpersonal variabilities yield superior detection of the emotional transition and state for some persons than others. We manually investigated the reason by considering 3 cases: best, worst, and average, to explore the association between everyday contexts and emotions of individuals. We selected persons 9, 2, and 14 as the best, worst, and average cases, respectively. We plotted the heatmaps of daily spatial contexts, activities, and emotional states over time for the worst, best, and average cases in Figures 7, 8, and 9, respectively. The 3 figures show that the majority of the data were collected when the persons were indoors or at home. For the worst case shown in Figure 7, the day-to-day activities and the emotional states of the person do not exhibit noticeable patterns over time. This might explain why the ML models were unable to capture a strong pattern of the contexts and emotions. On the other hand, for the best case shown in Figure 8, we observe clearer patterns in spatial context, activities, and emotional states over time despite a large amount of missing data. For example, in the best case, the person’s emotional state is *dissuaded* while lying down and *pleased* while watching television at home. The average case shown in Figure 9 exhibits some clear patterns of spatial context, activities, and emotional states over time. For example, a person reported *pleasure* mostly while being with friends and *dissuaded* or *discordant* while sleeping at home or outdoors. Therefore, regular patterns in lifestyle are important for the predictability of emotional state and transition detection, and this study showed that such patterns can be captured by leveraging ML algorithms and data acquired via smartphones and consumer-grade wearable devices.

### Limitations

There are limitations in this study. First, the results are on the basis of a relatively small data set from a small geographical area. In addition, the subjects lacked diversity in age and occupation as the majority were students and researchers at UCSD. Moreover, some spatiotemporal patterns may not have been captured in the data due to large amounts of missing values as well as limited data collection periods (maximum 9 days).

Owing to the small number of participants included in the data set, it was infeasible to hold a test set in conjunction with cross-validation for hyperparameter tuning. As a result, the classification performances reported in this paper are likely overestimated.

In addition, the emotional transition and state classes were highly imbalanced, where 1 or 2 classes of emotional states were absent in some persons’ data. Therefore, we suggest replicating our experiments on larger data sets obtained from diverse populations and geographic locations for longer periods of time.

Furthermore, the contextual labels that the classifications were based on were self-reported. Reliance on self-reported data is another limitation of the data set. We assumed that this self-reported information was correct in all cases. Hence, the results from this study partially depend on the accuracy of this self-reported information. To extend this study toward truly passive emotional monitoring, future research should explore eliminating self-reporting by predicting contextual information on the basis of raw sensor data.

Although mapping the emotions into the PAD system results in 3 dimensions, we focused only on the most dominant one. This simplified the rich, multidimensional information that the PAD system provided for making classification tasks more feasible. An alternative is to create a new emotion mapping system specifically for the ExtraSensory data set via clustering in the 3D space defined by PAD. This is a worthwhile future research direction.

Another major limitation is the absence of health-related information such as BMI, gender, age, and mental health biomarkers in the data. Therefore, further investigation is needed to shed light on the association between health status and emotional transition and state of persons.

### Conclusions

In this study, we explored the feasibility of detecting emotional transitions and states by applying ML techniques to daily data captured via smartphones and smartwatches. Our results established an association between emotional transition and state and contextual information. We also investigated the salient contextual variables influencing emotional states and transitions. The interpersonal variability in our results bolsters the need for further research on personalized prediction of emotional states and transitions. The findings of this study support the utility of passive data collection, reduced self-reporting, enhanced tracking of psychological well-being, self-awareness, self-management, and risk prediction and just-in-time interventions.

## Appendix

Multimedia Appendix 1 Dataset description and extended experimental results.

## Abbreviations

ANEW: Affective Norms for English Words
AUROC: area under the receiver operating characteristic
CB: CatBoost
LR: logistic regression
ML: machine learning
PAD: pleasure, arousal, dominance
RF: random forest
SHAP: shapley additive explanation
SVMSMOTE: Support Vector Machines Synthetic Minority Over-sampling Technique
UCSD: University of California, San Diego
XGB: XGBoost
