# Supplementary material for: Using Machine Learning and Smartphone and Smartwatch Data to Detect Emotional States and Transitions: Exploratory Study
Source: JMIR Mhealth Uhealth. 2020 Sep 29;8(9):e17818. doi: 10.2196/17818 (PMC7584158; doi:10.2196/17818)
Supplement: Multimedia Appendix 1 [file mhealth_v8i9e17818_app1.docx]

**Appendix**

Table 1. Mapping emotions to PAD system taken from the electronic supplementary martial

(<https://link.springer.com/article/10.3758/s13428-012-0314-x#SecESM1>)

| **Word** | **Pleasure** | **Arousal** | **Dominance** |
| --- | --- | --- | --- |
| Active | 6.47 | 5.62 | 6.32 |
| Afraid | 2.25 | 5.12 | 2.71 |
| Alert | 5.38 | 5.14 | 6.58 |
| Amused | 7.05 | 4.27 | 5.93 |
| Angry | 2.53 | 6.2 | 4.11 |
| Ashamed | 2.52 | 5.65 | 4.63 |
| Attentive | 6.43 | 4.37 | 6.62 |
| Bored | 2.95 | 3.65 | 4.96 |
| Calm | 6.89 | 1.67 | 7.44 |
| Crazy | 5.14 | 5.9 | 4.19 |
| Determined | 6 | 4.68 | 7.09 |
| Disgusted | 2.68 | 4.89 | 4.24 |
| Distressed | 3.38 | 6.28 | 4.16 |
| Dreamy | 7.52 | 4.9 | 6.28 |
| Energetic | 7.57 | 6.1 | 5.81 |
| Enthusiastic | 7.55 | 5.9 | 6.21 |
| Excited | 8.11 | 6.43 | 7.33 |
| Frustrated | 2.55 | 5.4 | 3.85 |
| Guilty | 3.09 | 4.65 | 4.5 |
| Happy | 8.47 | 6.05 | 7.21 |
| High | 5.77 | 4.1 | 5.76 |
| Hostile | 2.35 | 5.39 | 4.36 |
| Hungry | 3.54 | 5.6 | 4.59 |
| In emotional pain | 2 | 6.27 | 3.47 |
| In love | 8 | 5.36 | 5.92 |
| In physical pain | 2 | 6.27 | 3.47 |
| Inspired | 6.89 | 5.56 | 7.3 |
| Interested | 6.83 | 4.45 | 6.83 |
| Irritable | 2.85 | 6.37 | 4.23 |
| Jittery | 3.35 | 4.89 | 3.5 |
| Lonely | 2.67 | 4.37 | 3.33 |
| Nervous | 3.56 | 5.51 | 4.02 |
| Normal | 6.17 | 2.29 | 6.39 |
| Nostalgic | 6.68 | 4.37 | 5.05 |
| Optimistic | 7.45 | 4.19 | 7 |
| Physically sick | 2.29 | 4.67 | 2.84 |
| Proud | 7 | 5.55 | 7.09 |
| Romantic | 7.61 | 5.12 | 6.45 |
| Sad | 2.1 | 3.49 | 3.84 |
| Scared | 2.8 | 6.1 | 4.2 |
| Serious | 5.88 | 4.05 | 5.67 |
| Sexy | 7.42 | 6.8 | 6 |
| Sleepy | 4.36 | 3.04 | 4.56 |
| Stressed | 1.79 | 4.72 | 3.85 |
| Strong | 6.81 | 5.14 | 6.54 |
| Tired | 4.29 | 3.67 | 5.06 |
| Untroubled | 6.21 | 3.02 | 6.14 |
| Upset | 2.45 | 4.49 | 4.3 |
| Worried | 3.27 | 5.81 | 4.18 |

Table 2. List of Features

| **Motion Sensor Data** | |
| --- | --- |
| *Description of the prefix:*   - raw_acc: The ‘raw’ version of acceleration obtained from phone’s accelerometer. - proc_gyro: Processed version of gyroscope measurements obtained from phone’s gyroscope. - raw_magnet: The ‘raw’ magnetometer data from the phone as opposed to the bias-fixed data from OS - watch_acceleration: Accelerometer signals from the watch. - watch_heading: Heading from the compass on the watch. | |
| 1 | raw_acc:magnitude_stats:mean' |
| 2 | 'raw_acc:magnitude_stats:std' |
| 3 | 'raw_acc:magnitude_stats:moment3' |
| 4 | 'raw_acc:magnitude_stats:moment4' |
| 5 | 'raw_acc:magnitude_stats:percentile25' |
| 6 | 'raw_acc:magnitude_stats:percentile50' |
| 7 | 'raw_acc:magnitude_stats:percentile75' |
| 8 | 'raw_acc:magnitude_stats:value_entropy' |
| 9 | 'raw_acc:magnitude_stats:time_entropy' |
| 10 | 'raw_acc:magnitude_spectrum:log_energy_band0' |
| 11 | 'raw_acc:magnitude_spectrum:log_energy_band1' |
| 12 | 'raw_acc:magnitude_spectrum:log_energy_band2' |
| 13 | 'raw_acc:magnitude_spectrum:log_energy_band3' |
| 14 | 'raw_acc:magnitude_spectrum:log_energy_band4' |
| 15 | 'raw_acc:magnitude_spectrum:spectral_entropy' |
| 16 | 'raw_acc:magnitude_autocorrelation:period' |
| 17 | 'raw_acc:magnitude_autocorrelation:normalized_ac' |
| 18 | 'raw_acc:3d:mean_x' |
| 19 | 'raw_acc:3d:mean_y' |
| 20 | 'raw_acc:3d:mean_z' |
| 21 | 'raw_acc:3d:std_x' |
| 22 | 'raw_acc:3d:std_y' |
| 23 | 'raw_acc:3d:std_z' |
| 24 | 'raw_acc:3d:ro_xy' |
| 25 | 'raw_acc:3d:ro_xz' |
| 26 | 'raw_acc:3d:ro_yz' |
| 27 | 'proc_gyro:magnitude_stats:mean' |
| 28 | 'proc_gyro:magnitude_stats:std' |
| 29 | 'proc_gyro:magnitude_stats:moment3' |
| 30 | 'proc_gyro:magnitude_stats:moment4' |
| 31 | 'proc_gyro:magnitude_stats:percentile25' |
| 32 | 'proc_gyro:magnitude_stats:percentile50' |
| 33 | 'proc_gyro:magnitude_stats:percentile75' |
| 34 | 'proc_gyro:magnitude_stats:value_entropy' |
| 35 | 'proc_gyro:magnitude_stats:time_entropy' |
| 36 | 'proc_gyro:magnitude_spectrum:log_energy_band0' |
| 37 | 'proc_gyro:magnitude_spectrum:log_energy_band1' |
| 38 | 'proc_gyro:magnitude_spectrum:log_energy_band2' |
| 39 | 'proc_gyro:magnitude_spectrum:log_energy_band3' |
| 40 | 'proc_gyro:magnitude_spectrum:log_energy_band4' |
| 41 | 'proc_gyro:magnitude_spectrum:spectral_entropy' |
| 42 | 'proc_gyro:magnitude_autocorrelation:period' |
| 43 | 'proc_gyro:magnitude_autocorrelation:normalized_ac' |
| 44 | 'proc_gyro:3d:mean_x' |
| 45 | 'proc_gyro:3d:mean_y' |
| 46 | 'proc_gyro:3d:mean_z' |
| 47 | 'proc_gyro:3d:std_x' |
| 48 | 'proc_gyro:3d:std_y' |
| 49 | 'proc_gyro:3d:std_z' |
| 50 | 'proc_gyro:3d:ro_xy' |
| 51 | 'proc_gyro:3d:ro_xz' |
| 52 | 'proc_gyro:3d:ro_yz' |
| 53 | 'raw_magnet:magnitude_stats:mean' |
| 54 | 'raw_magnet:magnitude_stats:std' |
| 55 | 'raw_magnet:magnitude_stats:moment3' |
| 56 | 'raw_magnet:magnitude_stats:moment4' |
| 57 | 'raw_magnet:magnitude_stats:percentile25' |
| 58 | 'raw_magnet:magnitude_stats:percentile50' |
| 59 | 'raw_magnet:magnitude_stats:percentile75' |
| 60 | 'raw_magnet:magnitude_stats:value_entropy' |
| 61 | 'raw_magnet:magnitude_stats:time_entropy' |
| 62 | 'raw_magnet:magnitude_spectrum:log_energy_band0' |
| 63 | 'raw_magnet:magnitude_spectrum:log_energy_band1' |
| 64 | 'raw_magnet:magnitude_spectrum:log_energy_band2' |
| 65 | 'raw_magnet:magnitude_spectrum:log_energy_band3' |
| 66 | 'raw_magnet:magnitude_spectrum:log_energy_band4' |
| 67 | 'raw_magnet:magnitude_spectrum:spectral_entropy' |
| 68 | 'raw_magnet:magnitude_autocorrelation:period' |
| 69 | 'raw_magnet:magnitude_autocorrelation:normalized_ac' |
| 70 | 'raw_magnet:3d:mean_x' |
| 71 | 'raw_magnet:3d:mean_y' |
| 72 | 'raw_magnet:3d:mean_z' |
| 73 | 'raw_magnet:3d:std_x' |
| 74 | 'raw_magnet:3d:std_y' |
| 75 | 'raw_magnet:3d:std_z' |
| 76 | 'raw_magnet:3d:ro_xy' |
| 77 | 'raw_magnet:3d:ro_xz' |
| 78 | 'raw_magnet:3d:ro_yz' |
| 79 | 'raw_magnet:avr_cosine_similarity_lag_range0' |
| 80 | 'raw_magnet:avr_cosine_similarity_lag_range1' |
| 81 | 'raw_magnet:avr_cosine_similarity_lag_range2' |
| 82 | 'raw_magnet:avr_cosine_similarity_lag_range3' |
| 83 | 'raw_magnet:avr_cosine_similarity_lag_range4' |
| 84 | 'watch_acceleration:magnitude_stats:mean' |
| 85 | 'watch_acceleration:magnitude_stats:std' |
| 86 | 'watch_acceleration:magnitude_stats:moment3' |
| 87 | 'watch_acceleration:magnitude_stats:moment4' |
| 88 | 'watch_acceleration:magnitude_stats:percentile25' |
| 89 | 'watch_acceleration:magnitude_stats:percentile50' |
| 90 | 'watch_acceleration:magnitude_stats:percentile75' |
| 91 | 'watch_acceleration:magnitude_stats:value_entropy' |
| 92 | 'watch_acceleration:magnitude_stats:time_entropy' |
| 93 | 'watch_acceleration:magnitude_spectrum:log_energy_band0' |
| 94 | 'watch_acceleration:magnitude_spectrum:log_energy_band1' |
| 95 | 'watch_acceleration:magnitude_spectrum:log_energy_band2' |
| 96 | 'watch_acceleration:magnitude_spectrum:log_energy_band3' |
| 97 | 'watch_acceleration:magnitude_spectrum:log_energy_band4' |
| 98 | 'watch_acceleration:magnitude_spectrum:spectral_entropy' |
| 99 | 'watch_acceleration:magnitude_autocorrelation:period' |
| 100 | 'watch_acceleration:magnitude_autocorrelation:normalized_ac' |
| 101 | 'watch_acceleration:3d:mean_x' |
| 102 | 'watch_acceleration:3d:mean_y' |
| 103 | 'watch_acceleration:3d:mean_z' |
| 104 | 'watch_acceleration:3d:std_x' |
| 105 | 'watch_acceleration:3d:std_y' |
| 106 | 'watch_acceleration:3d:std_z' |
| 107 | 'watch_acceleration:3d:ro_xy' |
| 108 | 'watch_acceleration:3d:ro_xz' |
| 109 | 'watch_acceleration:3d:ro_yz' |
| 110 | 'watch_acceleration:spectrum:x_log_energy_band0' |
| 111 | 'watch_acceleration:spectrum:x_log_energy_band1' |
| 112 | 'watch_acceleration:spectrum:x_log_energy_band2' |
| 113 | 'watch_acceleration:spectrum:x_log_energy_band3' |
| 114 | 'watch_acceleration:spectrum:x_log_energy_band4' |
| 115 | 'watch_acceleration:spectrum:y_log_energy_band0' |
| 116 | 'watch_acceleration:spectrum:y_log_energy_band1' |
| 117 | 'watch_acceleration:spectrum:y_log_energy_band2' |
| 118 | 'watch_acceleration:spectrum:y_log_energy_band3' |
| 119 | 'watch_acceleration:spectrum:y_log_energy_band4' |
| 120 | 'watch_acceleration:spectrum:z_log_energy_band0' |
| 121 | 'watch_acceleration:spectrum:z_log_energy_band1' |
| 122 | 'watch_acceleration:spectrum:z_log_energy_band2' |
| 123 | 'watch_acceleration:spectrum:z_log_energy_band3' |
| 124 | 'watch_acceleration:spectrum:z_log_energy_band4' |
| 125 | 'watch_acceleration:relative_directions:avr_cosine_similarity_lag_range0' |
| 126 | 'watch_acceleration:relative_directions:avr_cosine_similarity_lag_range1' |
| 127 | 'watch_acceleration:relative_directions:avr_cosine_similarity_lag_range2' |
| 128 | 'watch_acceleration:relative_directions:avr_cosine_similarity_lag_range3' |
| 129 | 'watch_acceleration:relative_directions:avr_cosine_similarity_lag_range4' |
| 130 | 'watch_heading:mean_cos' |
| 131 | 'watch_heading:std_cos' |
| 132 | 'watch_heading:mom3_cos' |
| 133 | 'watch_heading:mom4_cos' |
| 134 | 'watch_heading:mean_sin' |
| 135 | 'watch_heading:std_sin' |
| 136 | 'watch_heading:mom3_sin' |
| 137 | 'watch_heading:mom4_sin' |
| 138 | 'watch_heading:entropy_8bins' |
| **Audio Sensor Data** | |
| *Description of the prefix:*   - audio_naive: Averages and standard deviations of the 13 MFCCs from the ~20sec recording windows of microphones. | |
| 139 | 'audio_naive:mfcc0:mean' |
| 140 | 'audio_naive:mfcc1:mean' |
| 141 | 'audio_naive:mfcc2:mean' |
| 142 | 'audio_naive:mfcc3:mean' |
| 143 | 'audio_naive:mfcc4:mean' |
| 144 | 'audio_naive:mfcc5:mean' |
| 145 | 'audio_naive:mfcc6:mean' |
| 146 | 'audio_naive:mfcc7:mean' |
| 147 | 'audio_naive:mfcc8:mean' |
| 148 | 'audio_naive:mfcc9:mean' |
| 149 | 'audio_naive:mfcc10:mean' |
| 150 | 'audio_naive:mfcc11:mean' |
| 151 | 'audio_naive:mfcc12:mean' |
| 152 | 'audio_naive:mfcc0:std' |
| 153 | 'audio_naive:mfcc1:std' |
| 154 | 'audio_naive:mfcc2:std' |
| 155 | 'audio_naive:mfcc3:std' |
| 156 | 'audio_naive:mfcc4:std' |
| 157 | 'audio_naive:mfcc5:std' |
| 158 | 'audio_naive:mfcc6:std' |
| 159 | 'audio_naive:mfcc7:std' |
| 160 | 'audio_naive:mfcc8:std' |
| 161 | 'audio_naive:mfcc9:std' |
| 162 | 'audio_naive:mfcc10:std' |
| 163 | 'audio_naive:mfcc11:std' |
| 164 | 'audio_naive:mfcc12:std' |
| 165 | 'audio_properties:max_abs_value' |
| 166 | 'audio_properties:normalization_multiplier' |
| **Location Data** | |
| *Description of the prefix:*   - location: Location services features that were extracted offline for every example from the sequence of latitude-longitude-altitude updates from the example’s minute. - location_quick_features: Location services features that were calculated on the phone when data was collected. | |
| 167 | 'location:num_valid_updates' |
| 168 | 'location:log_latitude_range', |
| 169 | 'location:log_longitude_range' |
| 170 | 'location:min_altitude', |
| 171 | 'location:max_altitude' |
| 172 | 'location:min_speed', |
| 173 | 'location:max_speed' |
| 174 | 'location:best_horizontal_accuracy' |
| 175 | 'location:best_vertical_accuracy' |
| 176 | 'location:diameter' |
| 177 | 'location:log_diameter' |
| 178 | 'location_quick_features:std_lat' |
| 179 | 'location_quick_features:std_long' |
| 180 | 'location_quick_features:lat_change' |
| 181 | 'location_quick_features:long_change' |
| 182 | 'location_quick_features:mean_abs_lat_deriv' |
| 183 | 'location_quick_features:mean_abs_long_deriv' |
| 184 | ‘cl_latitude’ |
| 185 | ‘cl_longitude’ |
| 186 | ‘geo_dist’ |
| **Phone State Data** | |
| *Description of the prefix:*   - discrete: These are binary indicators for the state of the phone. - lf_measurements: Various sensors that were recorded in low-frequency. | |
| 187 | 'discrete:app_state:is_active' |
| 188 | 'discrete:app_state:is_inactive' |
| 189 | 'discrete:app_state:is_background' |
| 190 | 'discrete:app_state:missing' |
| 191 | 'discrete:battery_plugged:is_ac' |
| 192 | 'discrete:battery_plugged:is_usb' |
| 193 | 'discrete:battery_plugged:is_wireless' |
| 194 | 'discrete:battery_plugged:missing' |
| 195 | 'discrete:battery_state:is_unknown' |
| 196 | 'discrete:battery_state:is_unplugged' |
| 197 | 'discrete:battery_state:is_not_charging' |
| 198 | 'discrete:battery_state:is_discharging' |
| 199 | 'discrete:battery_state:is_charging' |
| 200 | 'discrete:battery_state:is_full' |
| 201 | 'discrete:battery_state:missing' |
| 202 | 'discrete:on_the_phone:is_False' |
| 203 | 'discrete:on_the_phone:is_True' |
| 204 | 'discrete:on_the_phone:missing' |
| 205 | 'discrete:ringer_mode:is_normal' |
| 206 | 'discrete:ringer_mode:is_silent_no_vibrate' |
| 207 | 'discrete:ringer_mode:is_silent_with_vibrate' |
| 208 | 'discrete:ringer_mode:missing' |
| 209 | 'discrete:wifi_status:is_not_reachable' |
| 210 | 'discrete:wifi_status:is_reachable_via_wifi' |
| 211 | 'discrete:wifi_status:is_reachable_via_wwan' |
| 212 | 'discrete:wifi_status:missing' |
| 213 | 'lf_measurements:battery_level' |
| 214 | 'lf_measurements:screen_brightness' |
| **Environmental Data** | |
| *Description of the prefix:*   - lf_measurements: Various sensors that were recorded in low-frequency. | |
| 215 | 'lf_measurements:light' |
| 216 | 'lf_measurements:pressure' |
| 217 | 'lf_measurements:proximity_cm' |
| 218 | 'lf_measurements:proximity' |
| 219 | 'lf_measurements:relative_humidity' |
| 220 | 'lf_measurements:temperature_ambient' |
| **Temporal Data** | |
| *Description:*  Temporal features engineered from timestamps indicating minute of the hour, minute of the day (in 24 hours), hour of the day, day of the week, and time difference between two consecutive samples. | |
| 221 | ‘minute of the hour’ |
| 222 | ‘minute of the day’ |
| 223 | ‘hour of the day’ |
| 224 | ‘day of week’ |
| 225 | ‘time difference in minutes’ |
| **Contextual Data** | |
| *Description of the prefix:*   - All capitalized labels are the original labels user’s reported via interface of the mobile app. - Labels starting with 'FIX_' are the cleaned version of the corresponding labels, where the inconsistencies of the user reports were fixed by the researchers of the ExtraSensory dataset. - Labels starting with 'OR_' are the synthesized labels, which were created from a combination of other related labels. - Labels starting with with 'LOC_', are cleaned version of the corresponding labels that were fixed by researchers based on absolute location. | |
| 226 | label:LYING_DOWN' |
| 227 | 'label:SITTING' |
| 228 | 'label:FIX_walking' |
| 229 | 'label:FIX_running' |
| 230 | 'label:BICYCLING' |
| 231 | 'label:SLEEPING' |
| 232 | 'label:LAB_WORK' |
| 233 | 'label:IN_CLASS' |
| 234 | 'label:IN_A_MEETING' |
| 235 | 'label:LOC_main_workplace' |
| 236 | 'label:OR_indoors' |
| 237 | 'label:OR_outside' |
| 238 | 'label:IN_A_CAR' |
| 239 | 'label:ON_A_BUS' |
| 240 | 'label:DRIVE_-_I_M_THE_DRIVER' |
| 241 | 'label:DRIVE_-_I_M_A_PASSENGER' |
| 242 | 'label:LOC_home' |
| 243 | 'label:FIX_restaurant' |
| 244 | 'label:PHONE_IN_POCKET' |
| 245 | 'label:OR_exercise' |
| 246 | 'label:COOKING' |
| 247 | 'label:SHOPPING' |
| 248 | 'label:STROLLING' |
| 249 | 'label:DRINKING__ALCOHOL_' |
| 250 | 'label:BATHING_-_SHOWER' |
| 251 | 'label:CLEANING' |
| 252 | 'label:DOING_LAUNDRY' |
| 253 | 'label:WASHING_DISHES' |
| 254 | 'label:WATCHING_TV' |
| 255 | 'label:SURFING_THE_INTERNET' |
| 256 | 'label:AT_A_PARTY' |
| 257 | 'label:AT_A_BAR' |
| 258 | 'label:LOC_beach' |
| 259 | 'label:SINGING' |
| 260 | 'label:TALKING' |
| 261 | 'label:COMPUTER_WORK' |
| 262 | 'label:EATING' |
| 263 | 'label:TOILET' |
| 264 | 'label:GROOMING' |
| 265 | 'label:DRESSING' |
| 266 | 'label:AT_THE_GYM' |
| 267 | 'label:STAIRS_-_GOING_UP' |
| 268 | 'label:STAIRS_-_GOING_DOWN' |
| 269 | 'label:ELEVATOR' |
| 270 | 'label:OR_standing' |
| 271 | 'label:AT_SCHOOL' |
| 272 | 'label:PHONE_IN_HAND' |
| 273 | 'label:PHONE_IN_BAG' |
| 274 | 'label:PHONE_ON_TABLE' |
| 275 | 'label:WITH_CO-WORKERS' |
| 276 | 'label:WITH_FRIENDS' |

More detailed information on the features can be found in the following sources

1. The ExtraSensory dataset. <http://extrasensory.ucsd.edu/>
2. Vaizman Y, Ellis K, Lanckriet G. Recognizing detailed human context in-the-wild from smartphones and smartwatches. IEEE Pervasive Computing 2017;16(4): 62-74. [doi: [10.1109/MPRV.2017.3971131](https://doi.org/10.1109/MPRV.2017.3971131)]
3. Vaizman Y, Ellis K, Lanckriet G, Weibel N. Extrasensory app: Data collection in-the-wild with rich user interface to self-report behavior. Proceedings of the 2018 CHI Conference on Human Factors in Computing Systems; 2018 April:554; ACM. [doi: <https://doi.org/10.1145/3173574.3174128>]

Table 3: Hyper-parameter search grids for emotional transition detection

| ML Model | Hyper-parameters |
| --- | --- |
| Logistic Regression (LR) Classifier | param_grid = {'penalty' : ['l2'], \  'solver' : ['newton-cg', 'lbfgs', 'liblinear', 'sag', 'saga'],\  'max_iter':[100, 150, 200], 'class_weight':[None, 'balanced'] } |
| Random Forest (RF) Classifier | param_grid = {'criterion':['gini','entropy'], 'bootstrap': [True],\  'max_features':['log2', None],  'max_depth': np.arange(1, 30, 2),'n_estimators': [50, 100, 200, 300, 400]} |
| XGBoost (XGB) Classifier | param_grid = {"learning_rate": [0.05, 0.1, 0.15, 0.2], 'colsample_bytree':[.5, .75, 1],  'max_depth': np.arange(1, 20, 1),'n_estimators': [10, 20, 30, 50, 100, 200, 300, 400]} |
| CatBoost (CB) Classifier | param_grid = {"learning_rate": [0.05, 0.1, 0.15, 0.2],\  'depth': np.arange(1, 8, 1),'n_estimators': [10, 50, 100, 300, 500, 1000]} |
| Multi-layer Perceptron (MLP) Classifier | param_grid = {'hidden_layer_sizes' : [(50, 50, 50), (50, 100, 50), (20, 20, 20), (30, ), (50,),(100,)], \  'activation' : ['tanh', 'relu', 'logistic'],\  'max_iter':[50, 100, 150, 200, 300],\  'solver': ['sgd', 'adam', 'lbfgs'],\  'alpha': [0.0001, 0.001, 0.05]} |

Table 4: Hyper-parameter search grids for emotional state detection

| ML Model | Hyper-parameters |
| --- | --- |
| Logistic Regression (LR) Classifier | param_grid = {'penalty' : ['l2'], \  'solver' : ['newton-cg', 'lbfgs', 'liblinear', 'sag', 'saga'],\  'max_iter':[100, 150, 200], 'class_weight':[None, 'balanced'] } |
| Random Forest (RF) Classifier | param_grid = {'criterion':['gini','entropy'], 'bootstrap': [True],\  'max_features':['log2', None],  'max_depth': np.arange(1, 30, 2),'n_estimators': [50, 100, 200, 300, 400]} |
| XGBoost (XGB) Classifier | param_grid = {"learning_rate": [0.05, 0.1, 0.15, 0.2], 'colsample_bytree':[.5, .75, 1],  'max_depth': np.arange(1, 20, 1),'n_estimators': [10, 20, 30, 50, 100, 200, 300, 400]} |
| CatBoost (CB) Classifier | param_grid = {"learning_rate": [0.05, 0.1,0.15,0.2]} |
| Multi-layer Perceptron (MLP) Classifier | param_grid = {'hidden_layer_sizes' : [(50, 50, 50), (50, 100, 50), (20, 20, 20), (30, ), (50,),(100,)], \  'activation' : ['tanh', 'relu', 'logistic'],\  'max_iter':[50, 100, 150, 200, 300],\  'solver': ['sgd', 'adam', 'lbfgs'],\  'alpha': [0.0001, 0.001, 0.05]} |

Table 5: Results of the general models for emotional transition detection using different ML models and imbalance handling methods with 6-fold, leave-3-people-out cross-validation. Results are shown in Average (SD).

| **No imbalance handling** | **SMOTE** | **SVMSMOTE** |
| --- | --- | --- |
| Results for: Logistic Regression  .....................  Accuracy: 94.77% (1.64)  Balanced_accuracy: 60.59% (4.26)  Precision: 50.08% (10.55)  Recall: 22.32% (8.49)  Specificity: 98.85% (0.44)  ROC AUC: 90.50% (3.01)  F1 score: 29.89% (9.51)  ..................................................  Results for: RandomForest Classifier  .....................  Accuracy: 94.83% (1.47)  Balanced_accuracy: 56.54% (0.97)  Precision: 51.97% (10.16)  Recall: 13.85% (2.32)  Specificity: 99.23% (0.42)  ROC AUC: 89.07% (3.94)  F1 score: 21.44% (2.27)  ..................................................  Results for: MLP Classifier  .....................  Accuracy: 94.81% (1.63)  Balanced_accuracy: 53.53% (3.28)  Precision: 52.05% (8.59)  Recall: 7.50% (6.84)  Specificity: 99.57% (0.40)  ROC AUC: 88.16% (3.66)  F1 score: 11.91% (9.56)  ..................................................  Results for: XGBoost Classifier  .....................  Accuracy: 94.50% (1.54)  Balanced_accuracy: 66.92% (5.41)  Precision: 51.77% (16.98)  Recall: 36.03% (11.70)  Specificity: 97.81% (1.29)  ROC AUC: 89.72% (2.51)  F1 score: 38.85% (7.83)  ..................................................  Results for: CatBoost Classifier  .....................  Accuracy: 94.60% (1.58)  Balanced_accuracy: 65.02% (5.47)  Precision: 51.01% (12.13)  Recall: 31.98% (12.32)  Specificity: 98.05% (1.53)  ROC AUC: 89.24% (2.51)  F1 score: 36.46% (6.72)  .................................................. | Results for: Logistic Regression  .....................  Accuracy: 87.37% (1.02)  Balanced_accuracy: 83.36% (4.52)  Precision: 25.72% (5.08)  Recall: 78.74% (9.20)  Specificity: 87.98% (0.99)  ROC AUC: 90.33% (2.54)  F1 score: 38.18% (5.36)  ..................................................  Results for: RandomForest Classifier  .....................  Accuracy: 22.67% (21.84)  Balanced_accuracy: 55.42% (9.87)  Precision: 6.38% (3.06)  Recall: 91.95% (5.57)  Specificity: 18.88% (23.04)  ROC AUC: 48.25% (19.75)  F1 score: 11.77% (5.16)  ..................................................  Results for: MLP Classifier  .....................  Accuracy: 85.56% (3.11)  Balanced_accuracy: 81.90% (3.24)  Precision: 23.50% (5.52)  Recall: 77.75% (5.21)  Specificity: 86.04% (3.16)  ROC AUC: 89.62% (3.32)  F1 score: 35.61% (6.55)  ..................................................  Results for: XGBoost Classifier  .....................  Accuracy: 6.32% (2.09)  Balanced_accuracy: 50.40% (0.57)  Precision: 5.21% (1.62)  Recall: 99.54% (0.65)  Specificity: 1.25% (1.10)  ROC AUC: 69.40% (5.39)  F1 score: 9.86% (2.90)  ..................................................  Results for: CatBoost Classifier  .....................  Accuracy: 43.81% (33.73)  Balanced_accuracy: 57.29% (11.67)  Precision: 8.86% (4.44)  Recall: 72.38% (19.04)  Specificity: 42.20% (36.21)  ROC AUC: 66.50% (12.00)  F1 score: 14.83% (6.11)  .................................................. | Results for: Logistic Regression  .....................  Accuracy: 91.16% (1.24)  Balanced_accuracy: 80.76% (4.68)  Precision: 32.59% (5.47)  Recall: 69.05% (9.46)  Specificity: 92.48% (0.99)  ROC AUC: 90.26% (3.20)  F1 score: 43.63% (5.05)  ..................................................  Results for: RandomForest Classifier  .....................  Accuracy: 33.70% (28.16)  Balanced_accuracy: 58.75% (10.50)  Precision: 7.86% (4.75)  Recall: 86.49% (11.06)  Specificity: 31.00% (30.27)  ROC AUC: 66.65% (19.65)  F1 score: 13.81% (7.02)  ..................................................  Results for: MLP Classifier  .....................  Accuracy: 90.60% (1.93)  Balanced_accuracy: 81.28% (3.79)  Precision: 31.66% (6.15)  Recall: 70.82% (6.63)  Specificity: 91.75% (1.62)  ROC AUC: 90.35% (3.58)  F1 score: 43.29% (6.29)  ..................................................  Results for: XGBoost Classifier  .....................  Accuracy: 5.33% (1.46)  Balanced_accuracy: 50.09% (0.18)  Precision: 5.17% (1.58)  Recall: 100.00% (0.00)  Specificity: 0.17% (0.36)  ROC AUC: 60.49% (14.11)  F1 score: 9.80% (2.83)  ..................................................  Results for: CatBoost Classifier  .....................  Accuracy: 41.31% (33.75)  Balanced_accuracy: 60.56% (10.45)  Precision: 9.37% (4.73)  Recall: 82.27% (20.45)  Specificity: 38.84% (36.35)  ROC AUC: 74.77% (9.75)  F1 score: 15.78% (6.53)  ................................................. |

Table 6: Results of the general models for emotional state detection using different ML models and imbalance handling methods with 6-fold, leave-3-people-out cross-validation. Results are shown in Average (SD).

| **No imbalance handling** | **SMOTE** | **SVMSMOTE** |
| --- | --- | --- |
| Results for: Logistic Regression  .....................  Accuracy: 40.60% (9.50)  Balanced_accuracy: 22.83% (2.64)  Precision: 34.25% (5.26)  Recall: 38.86% (6.68)  Specificity: 63.37% (12.40)  ROC AUC: 60.23% (8.15)  F1 score: 30.60% (5.83)  ..................................................  Results for: RandomForest Classifier  .....................  Accuracy: 44.04% (14.42)  Balanced_accuracy: 21.84% (2.03)  Precision: 20.36% (7.50)  Recall: 29.40% (3.87)  Specificity: 71.23% (4.73)  ROC AUC: 55.21% (4.31)  F1 score: 19.20% (5.19)  ..................................................  Results for: MLP Classifier  .....................  Accuracy: 44.84% (14.73)  Balanced_accuracy: 21.67% (2.36)  Precision: 44.84% (14.73)  Recall: 100.00% (0.00)  Specificity: 0.00% (0.00)  ROC AUC: 48.41% (8.17)  F1 score: 60.30% (15.86)  ..................................................  Results for: XGBoost Classifier  .....................  Accuracy: 39.39% (12.70)  Balanced_accuracy: 23.42% (1.65)  Precision: 20.75% (3.28)  Recall: 25.90% (5.05)  Specificity: 76.77% (5.10)  ROC AUC: 53.96% (5.98)  F1 score: 17.75% (4.53)  ..................................................  Results for: CatBoost Classifier  .....................  Accuracy: 44.10% (13.93)  Balanced_accuracy: 24.32% (3.23)  Precision: 29.52% (11.39)  Recall: 33.29% (8.57)  Specificity: 72.40% (3.62)  ROC AUC: 58.58% (6.97)  F1 score: 25.45% (10.72)  .................................................. | Results for: Logistic Regression  .....................  Accuracy: 31.78% (3.08)  Balanced_accuracy: 28.31% (6.85)  Precision: 27.59% (3.22)  Recall: 26.07% (5.30)  Specificity: 83.15% (0.90)  ROC AUC: 59.89% (4.73)  F1 score: 23.34% (3.81)  ..................................................  Results for: RandomForest Classifier  .....................  Accuracy: 41.21% (11.51)  Balanced_accuracy: 22.77% (2.21)  Precision: 24.08% (5.92)  Recall: 24.68% (3.59)  Specificity: 77.86% (2.59)  ROC AUC: 58.11% (4.89)  F1 score: 19.06% (4.61)  ..................................................  Results for: MLP Classifier  .....................  Accuracy: 40.97% (16.70)  Balanced_accuracy: 21.03% (3.29)  Precision: 33.96% (17.46)  Recall: 62.58% (27.46)  Specificity: 36.41% (26.38)  ROC AUC: 47.46% (7.08)  F1 score: 42.52% (21.92)  ..................................................  Results for: XGBoost Classifier  .....................  Accuracy: 43.14% (13.80)  Balanced_accuracy: 22.64% (2.64)  Precision: 21.17% (5.13)  Recall: 30.76% (6.45)  Specificity: 71.21% (4.17)  ROC AUC: 56.99% (7.03)  F1 score: 20.51% (7.29)  ..................................................  Results for: CatBoost Classifier  .....................  Accuracy: 43.61% (14.00)  Balanced_accuracy: 22.77% (2.78)  Precision: 27.31% (8.84)  Recall: 40.63% (7.12)  Specificity: 61.50% (8.29)  ROC AUC: 56.85% (5.33)  F1 score: 25.74% (5.74)  .................................................. | Results for: Logistic Regression  .....................  Accuracy: 32.61% (2.65)  Balanced_accuracy: 30.66% (8.82)  Precision: 26.48% (2.32)  Recall: 28.27% (7.76)  Specificity: 82.93% (0.76)  ROC AUC: 60.55% (3.41)  F1 score: 23.04% (2.82)  ..................................................  Results for: RandomForest Classifier  .....................  Accuracy: 38.99% (8.71)  Balanced_accuracy: 23.27% (2.43)  Precision: 25.33% (2.20)  Recall: 25.94% (2.08)  Specificity: 76.93% (2.63)  ROC AUC: 55.43% (4.37)  F1 score: 21.81% (3.24)  ..................................................  Results for: MLP Classifier  .....................  Accuracy: 34.24% (17.81)  Balanced_accuracy: 17.85% (4.63)  Precision: 29.04% (15.81)  Recall: 45.56% (26.76)  Specificity: 47.74% (22.16)  ROC AUC: 49.46% (7.23)  F1 score: 32.78% (20.85)  ..................................................  Results for: XGBoost Classifier  .....................  Accuracy: 29.63% (15.13)  Balanced_accuracy: 24.17% (4.63)  Precision: 25.15% (5.78)  Recall: 25.40% (6.51)  Specificity: 78.49% (1.52)  ROC AUC: 54.10% (6.63)  F1 score: 19.06% (6.91)  ..................................................  Results for: CatBoost Classifier  .....................  Accuracy: 39.52% (13.24)  Balanced_accuracy: 23.32% (2.91)  Precision: 27.18% (10.23)  Recall: 27.81% (5.77)  Specificity: 75.36% (4.66)  ROC AUC: 56.83% (6.14)  F1 score: 21.20% (6.95)  .................................................. |

Table 7: Results of the personalized models for emotional transition detection using different ML models and imbalance handling methods using 5-fold stratified cross-validation. Results are shown in Average (SD).

| **No imbalance handling** | **SMOTE** | **SVMSMOTE** |
| --- | --- | --- |
| Results for: Logistic Regression  .....................  Accuracy: 95.36% (1.95)  Balanced_accuracy: 64.23% (9.42)  Precision: 50.36% (26.66)  Recall: 29.49% (19.26)  Specificity: 98.96% (0.93)  ROC AUC: 87.31% (13.23)  F1 score: 35.12% (20.47)  ..................................................  Results for: RandomForest Classifier  .....................  Accuracy: 93.49% (4.73)  Balanced_accuracy: 66.88% (10.08)  Precision: 49.97% (21.23)  Recall: 37.12% (20.78)  Specificity: 96.65% (4.91)  ROC AUC: 88.01% (5.67)  F1 score: 36.40% (17.87)  ..................................................  Results for: MLP Classifier  .....................  Accuracy: 94.78% (2.46)  Balanced_accuracy: 65.65% (12.36)  Precision: 40.60% (26.71)  Recall: 33.10% (25.12)  Specificity: 98.19% (1.35)  ROC AUC: 80.27% (18.56)  F1 score: 34.65% (25.38)  ..................................................  Results for: XGBoost Classifier  .....................  Accuracy: 94.82% (2.55)  Balanced_accuracy: 65.91% (7.93)  Precision: 52.34% (17.22)  Recall: 33.71% (15.69)  Specificity: 98.12% (1.42)  ROC AUC: 87.84% (6.81)  F1 score: 38.00% (15.92)  ..................................................  Results for: CatBoost Classifier  .....................  Accuracy: 94.29% (3.17)  Balanced_accuracy: 66.89% (8.97)  Precision: 48.67% (18.09)  Recall: 36.45% (18.10)  Specificity: 97.34% (2.33)  ROC AUC: 87.62% (7.04)  F1 score: 38.34% (17.07)  .................................................. | Results for: Logistic Regression  .....................  Accuracy: 90.61% (5.28)  Balanced_accuracy: 80.72% (5.59)  Precision: 37.91% (17.90)  Recall: 69.75% (9.51)  Specificity: 91.69% (5.35)  ROC AUC: 89.53% (5.65)  F1 score: 45.74% (13.96)  ..................................................  Results for: RandomForest Classifier  .....................  Accuracy: 89.06% (8.61)  Balanced_accuracy: 65.80% (8.28)  Precision: 31.61% (16.37)  Recall: 40.11% (18.79)  Specificity: 91.49% (9.25)  ROC AUC: 83.12% (7.99)  F1 score: 29.61% (12.81)  ..................................................  Results for: MLP Classifier  .....................  Accuracy: 90.23% (7.36)  Balanced_accuracy: 72.15% (6.01)  Precision: 37.77% (15.53)  Recall: 52.01% (14.85)  Specificity: 92.29% (7.99)  ROC AUC: 84.88% (8.58)  F1 score: 39.01% (11.26)  ..................................................  Results for: XGBoost Classifier  .....................  Accuracy: 84.23% (10.90)  Balanced_accuracy: 74.17% (7.71)  Precision: 29.45% (12.18)  Recall: 63.19% (14.22)  Specificity: 85.16% (11.51)  ROC AUC: 87.11% (7.06)  F1 score: 36.23% (11.49)  ..................................................  Results for: CatBoost Classifier  .....................  Accuracy: 89.37% (10.22)  Balanced_accuracy: 69.18% (8.63)  Precision: 36.15% (18.55)  Recall: 46.68% (14.14)  Specificity: 91.68% (10.57)  ROC AUC: 85.87% (10.92)  F1 score: 36.32% (15.31)  .................................................. | Results for: Logistic Regression  .....................  Accuracy: 92.38% (3.18)  Balanced_accuracy: 78.54% (7.90)  Precision: 38.25% (12.92)  Recall: 63.11% (15.08)  Specificity: 93.97% (2.67)  ROC AUC: 88.67% (7.18)  F1 score: 46.29% (12.70)  ..................................................  Results for: RandomForest Classifier  .....................  Accuracy: 90.31% (7.17)  Balanced_accuracy: 66.80% (5.93)  Precision: 34.29% (17.13)  Recall: 40.77% (13.49)  Specificity: 92.82% (7.63)  ROC AUC: 85.08% (7.20)  F1 score: 32.92% (12.48)  ..................................................  Results for: MLP Classifier  .....................  Accuracy: 91.60% (6.07)  Balanced_accuracy: 71.61% (9.03)  Precision: 39.09% (19.82)  Recall: 49.39% (20.15)  Specificity: 93.83% (6.68)  ROC AUC: 82.05% (9.77)  F1 score: 40.32% (16.19)  ..................................................  Results for: XGBoost Classifier  .....................  Accuracy: 89.54% (6.06)  Balanced_accuracy: 75.86% (8.52)  Precision: 35.69% (13.32)  Recall: 60.76% (16.37)  Specificity: 90.97% (6.03)  ROC AUC: 87.74% (6.63)  F1 score: 41.85% (13.46)  ..................................................  Results for: CatBoost Classifier  .....................  Accuracy: 92.34% (5.01)  Balanced_accuracy: 70.28% (8.06)  Precision: 42.37% (14.18)  Recall: 45.82% (15.17)  Specificity: 94.75% (4.53)  ROC AUC: 88.73% (6.24)  F1 score: 40.44% (13.88)  .................................................. |

Table 8: Results of the personalized models for emotional state detection using different ML models and imbalance handling methods with 5-fold stratified cross-validation. Results are shown in Average (SD).

| **No imbalance handling** | **SMOTE** | **SVMSMOTE** |
| --- | --- | --- |
| Results for: Logistic Regression  .....................  Accuracy: 77.83% (10.20)  Balanced_accuracy: 58.40% (19.34)  Precision: 75.80% (11.10)  Recall: 71.64% (15.36)  Specificity: 82.22% (21.26)  ROC AUC: 90.07% (6.90)  F1 score: 70.68% (13.24)  ..................................................  Results for: RandomForest Classifier  .....................  Accuracy: 80.92% (10.69)  Balanced_accuracy: 67.85% (14.10)  Precision: 82.11% (8.67)  Recall: 71.96% (10.87)  Specificity: 89.83% (6.58)  ROC AUC: 93.74% (5.62)  F1 score: 72.46% (12.07)  ..................................................  Results for: MLP Classifier  .....................  Accuracy: 79.16% (12.79)  Balanced_accuracy: 64.82% (23.11)  Precision: 73.61% (16.19)  Recall: 74.34% (15.05)  Specificity: 82.77% (21.62)  ROC AUC: 85.89% (16.82)  F1 score: 71.84% (16.25)  ..................................................  Results for: XGBoost Classifier  .....................  Accuracy: 82.73% (7.18)  Balanced_accuracy: 69.51% (12.18)  Precision: 82.49% (7.32)  Recall: 74.54% (7.70)  Specificity: 90.48% (5.64)  ROC AUC: 94.81% (2.96)  F1 score: 74.87% (8.45)  ..................................................  Results for: CatBoost Classifier  .....................  Accuracy: 86.53% (8.08)  Balanced_accuracy: 74.92% (13.55)  Precision: 87.03% (9.37)  Recall: 77.88% (11.35)  Specificity: 92.92% (4.80)  ROC AUC: 96.33% (3.26)  F1 score: 79.48% (11.38)  .................................................. | Results for: Logistic Regression  .....................  Accuracy: 74.02% (11.00)  Balanced_accuracy: 76.21% (9.76)  Precision: 66.35% (13.60)  Recall: 76.21% (9.76)  Specificity: 91.10% (3.44)  ROC AUC: 91.21% (5.12)  F1 score: 67.67% (13.46)  ..................................................  Results for: RandomForest Classifier  .....................  Accuracy: 78.82% (9.98)  Balanced_accuracy: 69.65% (14.84)  Precision: 76.86% (10.69)  Recall: 73.57% (11.62)  Specificity: 89.89% (6.61)  ROC AUC: 92.88% (5.51)  F1 score: 71.10% (13.09)  ..................................................  Results for: MLP Classifier  .....................  Accuracy: 80.13% (12.89)  Balanced_accuracy: 76.80% (11.06)  Precision: 74.41% (14.34)  Recall: 77.19% (10.79)  Specificity: 92.34% (4.37)  ROC AUC: 92.67% (6.37)  F1 score: 73.52% (14.42)  ..................................................  Results for: XGBoost Classifier  .....................  Accuracy: 81.12% (8.92)  Balanced_accuracy: 72.80% (13.58)  Precision: 77.61% (11.06)  Recall: 75.75% (12.30)  Specificity: 91.47% (5.01)  ROC AUC: 94.49% (3.54)  F1 score: 73.13% (13.05)  ..................................................  Results for: CatBoost Classifier  .....................  Accuracy: 84.29% (9.47)  Balanced_accuracy: 74.78% (14.95)  Precision: 83.41% (9.76)  Recall: 77.28% (12.53)  Specificity: 92.11% (5.51)  ROC AUC: 96.06% (3.47)  F1 score: 77.37% (12.84)  .................................................. | Results for: Logistic Regression  .....................  Accuracy: 74.69% (10.70)  Balanced_accuracy: 75.01% (11.12)  Precision: 67.03% (13.53)  Recall: 75.38% (10.49)  Specificity: 91.05% (3.82)  ROC AUC: 90.93% (5.32)  F1 score: 68.23% (13.18)  ..................................................  Results for: RandomForest Classifier  .....................  Accuracy: 78.21% (10.78)  Balanced_accuracy: 70.72% (13.92)  Precision: 75.94% (12.02)  Recall: 73.30% (11.91)  Specificity: 90.19% (5.67)  ROC AUC: 92.93% (5.27)  F1 score: 70.73% (13.34)  ..................................................  Results for: MLP Classifier  .....................  Accuracy: 80.04% (13.80)  Balanced_accuracy: 75.75% (13.79)  Precision: 73.89% (15.37)  Recall: 76.76% (12.81)  Specificity: 92.09% (5.19)  ROC AUC: 92.12% (7.20)  F1 score: 73.46% (15.62)  ..................................................  Results for: XGBoost Classifier  .....................  Accuracy: 81.54% (8.90)  Balanced_accuracy: 74.50% (12.03)  Precision: 77.71% (11.09)  Recall: 77.23% (10.36)  Specificity: 91.96% (4.71)  ROC AUC: 94.51% (3.39)  F1 score: 74.51% (12.05)  ..................................................  Results for: CatBoost Classifier  .....................  Accuracy: 85.27% (9.64)  Balanced_accuracy: 77.73% (13.34)  Precision: 84.44% (9.83)  Recall: 80.00% (10.74)  Specificity: 93.15% (4.84)  ROC AUC: 96.26% (3.77)  F1 score: 79.47% (12.00)  .................................................. |
